# Metabolomic profiling and identification of potential biomarkers of highly pathogenic avian influenza (H5N1) in chicken

**DOI:** 10.3389/fcimb.2025.1540290

**Published:** 2025-10-07

**Authors:** Althaf Mohammed Kadamthodi, Anuradha Panwar, Akhila Hosur Shrungeswara, Periyasamy Vijayakumar, Thottethodi Subrahmanya Keshava Prasad, Ashwin Ashok Raut, Anamika Mishra

**Affiliations:** ^1^ Pathogenomics Lab, Indian Council of Agricultural Research (ICAR) – National Institute of High Security Animal Diseases, Bhopal, Madhya Pradesh, India; ^2^ Center for Systems Biology and Molecular Medicine [An ICMR-Collaborating Centre of Excellence 2024–2029 (ICMR CCoE 2024)], Yenepoya Research Centre, Yenepoya (Deemed to be University), Mangalore, Karnataka, India; ^3^ Department of Animal Genetics and Breeding, Veterinary College and Research Institute, Tamil Nadu Veterinary and Animal Sciences University, Salem, Tamil Nadu, India

**Keywords:** metabolomics, H5N1, Sphingolipid metabolism, tryptophan metabolism, SPF chicken

## Abstract

**Introduction:**

Highly Pathogenic Avian Influenza (HPAI) H5N1 is a significant zoonotic pathogen with the potential to cause pandemics. Its high prevalence and mortality rates in poultry, along with a recent expansion in host range, underscore the urgent need to understand the molecular mechanisms underlying its pathogenesis and host-pathogen interactions. Metabolomics, the comprehensive study of small-molecule metabolites within biological systems, offers a promising approach to unravel these mechanisms and aid in the development of effective control strategies against HPAI H5N1.

**Methods:**

To investigate the metabolomic alterations associated with HPAI H5N1 infection, serum and lung samples were collected from specific pathogen-free (SPF) chickens that were either infected with HPAI H5N1 or mock-infected as controls. Metabolomic profiling was performed using liquid chromatography-tandem mass spectrometry (LC-MS/MS) under both positive and negative ionization modes. The resulting data were analyzed to identify metabolites that were significantly altered in response to infection.

**Results:**

The metabolomic analysis revealed substantial changes in both lung and serum samples following HPAI H5N1 infection. Specifically, 31 and 13 altered metabolites were identified in the lung, and 22 and 15 in the serum, under positive and negative ionization modes, respectively. Notably, key metabolites such as sphingosine, psychosine sulfate, and L-serine, which are known to influence viral endocytosis and cell signaling, were significantly altered in infected chickens.

**Discussion:**

The observed changes in sphingolipid and tryptophan metabolism provide insights into the mechanisms underlying lung and central nervous system (CNS) pathology associated with HPAI H5N1 infection. This study represents the first comprehensive metabolomic profiling of HPAI H5N1-infected chickens, offering valuable information for the development of novel therapeutics and control strategies. The identification of specific metabolite alterations may guide future research aimed at mitigating the impact of this highly pathogenic virus.

## 1 Introduction

Influenza viruses, part of the Orthomyxoviridae family and the genus Influenza virus, are RNA viruses characterized by a segmented, single-stranded, negative-sense genome (Cargnin Faccin and Perez, 2024). These viruses are classified into four types: A (IAV), B (IBV), C (ICV), and D (IDV). IAV, in particular, is a pathogen of significant clinical importance, posing a considerable threat to both the poultry and public health (Zhang et al., 2024b). Avian influenza viruses (AIVs) are categorized based on their impact on chicken, as determined by the intravenous pathogenicity index (IVPI) test, into highly pathogenic avian influenza viruses (HPAIV) and low pathogenic avian influenza viruses (LPAIV) (Liu et al., 2023). Recently, the spread of HPAIV strains such as H5N1, H7N9, and H5N8 across different host species has become a serious public health concern (Sutton, 2018). Among these, H5N1 is particularly notable due to its high pathogenicity, leading to significant mortality in both chicken and humans (Kim et al., 2023). The HPAIV subtype H5N1 is already panzootic in poultry, causing severe economic impacts. It continues to cross species barriers, infecting humans and other mammals, often with fatal outcomes. Additionally, avian influenza viruses, including H5N1, are known to mutate rapidly. If a strain were to acquire the ability for sustained human-to-human transmission, it could lead to a pandemic situation similar to seasonal influenza but with potentially more severe consequences (Yamaji et al., 2020). Therefore, a deeper understanding of the host-pathogen interaction of HPAI H5N1 is crucial, given its prevalence, ability to infect humans, and potential for mutation. For this purpose, metabolomics is a superior tool as it offers a more effective means of understanding disease progression compared to other omics techniques, such as genomics, transcriptomics, and proteomics, due to its close association with phenotype and real-time biological processes.

Metabolomics involves the study of small chemical compounds (< 1500 atomic mass unit) produced or utilized within a biological system, including both primary and secondary metabolites. When performed accurately, metabolomics provides an unbiased analysis of a diverse range of small-molecule metabolites, collectively known as the metabolome, within a specific biological system under defined conditions (Snowden et al., 2012). This approach is useful for characterizing underlying pathological mechanisms and, more broadly, for monitoring and understanding phenotypic variations (Beale et al., 2019). Metabolomics can be conducted using either targeted or non-targeted approaches (Bingol, 2018). In this study we have utilized non-targeted approach.

Several studies have utilized metabolomic analysis to investigate influenza viruses (Tisoncik-Go et al., 2016; Cui et al., 2016; Milner et al., 2014, 2015; Chandler et al., 2016) primarily using mice and ferrets as experimental models. Zhang et al., 2024b, conducted metabolomic profiling of the H9N2 avian influenza virus in DF-1 cells. However, no metabolomic studies have been conducted on H5N1, particularly in chicken hosts. In this study, we are performing metabolomic profiling and identifying metabolomic markers following HPAI H5N1 infection in chicken.

## 2 Materials and methods

### 2.1 Ethics statement

The animal experiments were carried out at the Biosafety level 3 + containment facility ICAR-National Institute of High Security Animal Diseases, Bhopal, India, as per the guidelines of Institutional Animal Ethics Committee and Committee for the Purpose of Control and Supervision of Experiments on Animals (CPCSEA), Ministry of Environment and Forests, Govt. of India (Approval no. 125/IAEC/NIHSAD/21).

### 2.2 Animal experiment

Six, six-week-old, specific pathogen-free (SPF) chicken, were divided into two groups of three each. The first group was intranasally inoculated with 10^6^ EID_50_ of A/duck/India/02CA10/2011/Agartala avian influenza virus, while the control group received an intranasal inoculation of phosphate buffered saline (PBS). At 12 h post-inoculation, the birds were slightly dull and were starting to show a tendency to avoid physical activity. They were euthanized by cervical dislocation, and blood as well as lung tissue was collected. Lung samples were rinsed 3–4 times by immersing in ample volumes of triple solvent mixture (acetonitrile: methanol: water in a 2:2:1 ratio) to washout blood, divided into triplicates and then stored in triple solvent overnight at -20°C to inactivate the virus in the samples. Similarly, serum was combined with an equal volume of the triple solvent divided into three technical triplicates and kept at -20°C (Hu et al., 2021).Virus inactivation was confirmed by inoculation in embryonated chicken eggs. The RNA extracted from lungs and serum samples collected from the infected birds were screened using RT-qPCR for confirmation of presence of infection.

### 2.3 Metabolite extraction

Each sample was made into three technical replicates to ensure reliability and reproducibility of results, resulting in a total of 18 sample sets each for lung and serum. Metabolites were extracted from lung tissue and serum samples according to the protocol outlined by Lau et al. (2015). Lung tissue samples (50 mg each) were weighed, flash-frozen in liquid nitrogen, and ground into a fine powder using a mortar and pestle. Subsequently, 1 mL of triple solvent (acetonitrile:methanol:water in a 2:2:1 ratio) was added to the powdered tissue, and the mixture was sonicated for 3 minutes using a probe sonicator (Q Sonica, Parmer). For the serum samples, 25 μL of serum (inactivated serum mixture) was mixed with 450 μL of the triple solvent mixture, briefly vortexed, and then sonicated in a water bath. All samples, including lung tissue and serum, were stored overnight at -20°C. The following day, the samples were centrifuged at 12,000 × g for 15 minutes at 4°C to separate the supernatants, which were then collected and dried under vacuum using a SpeedVac. The dried residues were reconstituted in 250 μL of 0.1% formic acid, thoroughly mixed, and prepared for mass spectrometry analysis.

### 2.4 LC-MS/MS analysis

Metabolite extracts were analyzed using liquid chromatography followed by tandem mass spectrometry (LC-MS/MS) on a QTRAP 6500 mass spectrometer (AB Sciex) coupled with an Agilent 1290 Infinity II liquid chromatography system, equipped with a C18 Kinetex column (2.1 X 150 mm, 1.7μm). Data acquisition was performed using Analyst software version 1.6.3, with the Analyst Device Driver used to configure analysis parameters. Metabolite separation was achieved with a 25-minute Liquid Chromatography method, as outlined in **Supplementary Table 1**. Solvent A consisted of 0.1% formic acid in LC-MS grade water, while solvent B was 0.1% formic acid in 90% acetonitrile. The flow rate was set to 0.250 mL/min.

Mass spectrometry data was acquired using the Information-Dependent Acquisition (IDA) method in low mass mode. The IDA method was built using the EMS (enhanced mass spectra) to EPI (enhanced product ion) modes. The top five spectra from the EMS mode were selected for further analysis in the EPI (MS/MS) mode, utilizing high-energy collision-induced dissociation (CID). Metabolite data was collected in both positive and negative polarities, with voltages of 4500 V and -4500 V, respectively, and a probe temperature of 450°C. Compound parameters included a declustering potential (DP) of ±100 V and a collision energy (CE) of ±40 V. All resulting files in.wiff format were analyzed for the feature.

### 2.5 Data analysis

The LC-MS/MS results in.wiff format were converted to.mzML format using the ProteoWizard MSConvert tool. The.mzML raw data were then processed in MZMine 2.53 (Schmid et al., 2023).Feature detection was performed at both MS and MS/MS level, in centroid mode. The noise level greater than 1.0E1 was set to MS/MS level. This was followed by peak extension and chromatogram deconvolution, and isotope peak grouping. For replicate runs of infected and control samples, the deisotoped features were aligned using the Join-Aligner algorithm, gaps were filled, and duplicate peaks were removed. The.mgf files containing precursor and fragment information were used for metabolite assignment through fragment-level matches using the in-house MS2Compound tool (https://sourceforge.net/projects/ms2compound/). Metabolites from the Human Metabolite Database (HMDB) (https://hmdb.ca/) served as the backend database for identification. We selected HMDB because, at present, no dedicated metabolomics database exists specifically for chickens, as the field is still developing. However, many metabolites are highly conserved across species, and core metabolic pathways (such as glycolysis, the TCA cycle, and lipid metabolism) are shared among vertebrates. This makes HMDB a valuable and widely accepted resource for chicken metabolomic analysis. In fact, several poultry studies have successfully employed HMDB for metabolite identification despite the absence of a chicken-specific database (Wang et al., 2024; Tang et al., 2021; Zhang et al., 2024a). In this study, MS2Compound matched metabolites to precursor m/z values based on their charge states and corresponding adducts ([M+H]+ for positive mode and [M–H]– for negative mode). Metabolite assignment having highest rank and mS-score to a particular m/z considered for further downstream analysis. Following this, exogenous metabolites such as drugs, environmental contaminants, exclusive plant metabolites etc have been removed from further analysis.

### 2.6 Statistical analysis

Statistical analysis was performed using MetaboAnalyst 6.0 (https://www.metaboanalyst.ca/). Initially, Principal Component Analysis (PCA), an unsupervised method, was conducted to assess overall sample clustering. This was followed by a supervised analysis using Orthogonal Partial Least Square Discriminant Analysis (OPLS-DA), where metric values were obtained to evaluate the reliability of the experimental model. In the OPLS-DA analysis, Variable Importance in Projection (VIP) scores were calculated for all metabolites, with only those having VIP ≥ 1 retained for further analysis. To identify significantly differential metabolites between infected and control samples, t-tests and fold change analysis were performed, with thresholds set at p ≤ 0.05 for the t-test and 1.2 for fold change.

### 2.7 Pathway enrichment analysis

Pathway enrichment analysis was performed using MetaboAnalyst 6.0 (https://www.metaboanalyst.ca/). For this analysis, the combined list of significantly differential metabolites from both positive and negative ionization modes was used. Pathways with a p-value ≤ 0.05 were identified as significantly enriched.

### 2.8 Metabolomic marker identification

To identify potential metabolomic markers, receiver operating characteristic (ROC) curve analysis was performed on the significantly differential metabolites, and the Area Under the Curve (AUC) values were obtained for each metabolite. AUC values between 0.7 and 0.8 are considered acceptable, those between 0.8 and 0.9 are deemed excellent, and values above 0.9 are regarded as outstanding (Hosmer and Lemeshow, 2013). In this study, metabolites with AUC values greater than 0.7 were considered as potential metabolomic markers. From this list, markers were proposed based on their contribution to significant enriched pathways and existing evidence linking them to influenza A virus.

## 3 Results

### 3.1 Lung metabolomic profiling

LC-MS/MS-based metabolomic profiling of lung samples identified 3,602 aligned peaks in positive ionization mode and 2,678 in negative ionization mode using MZMine 2.53. The MS2Compound search assigned 851 metabolites in positive and 443 in negative ionization mode. After excluding exogenous metabolites, 196 metabolites in positive and 90 in negative ionization mode were retained.

**Figure 1:**
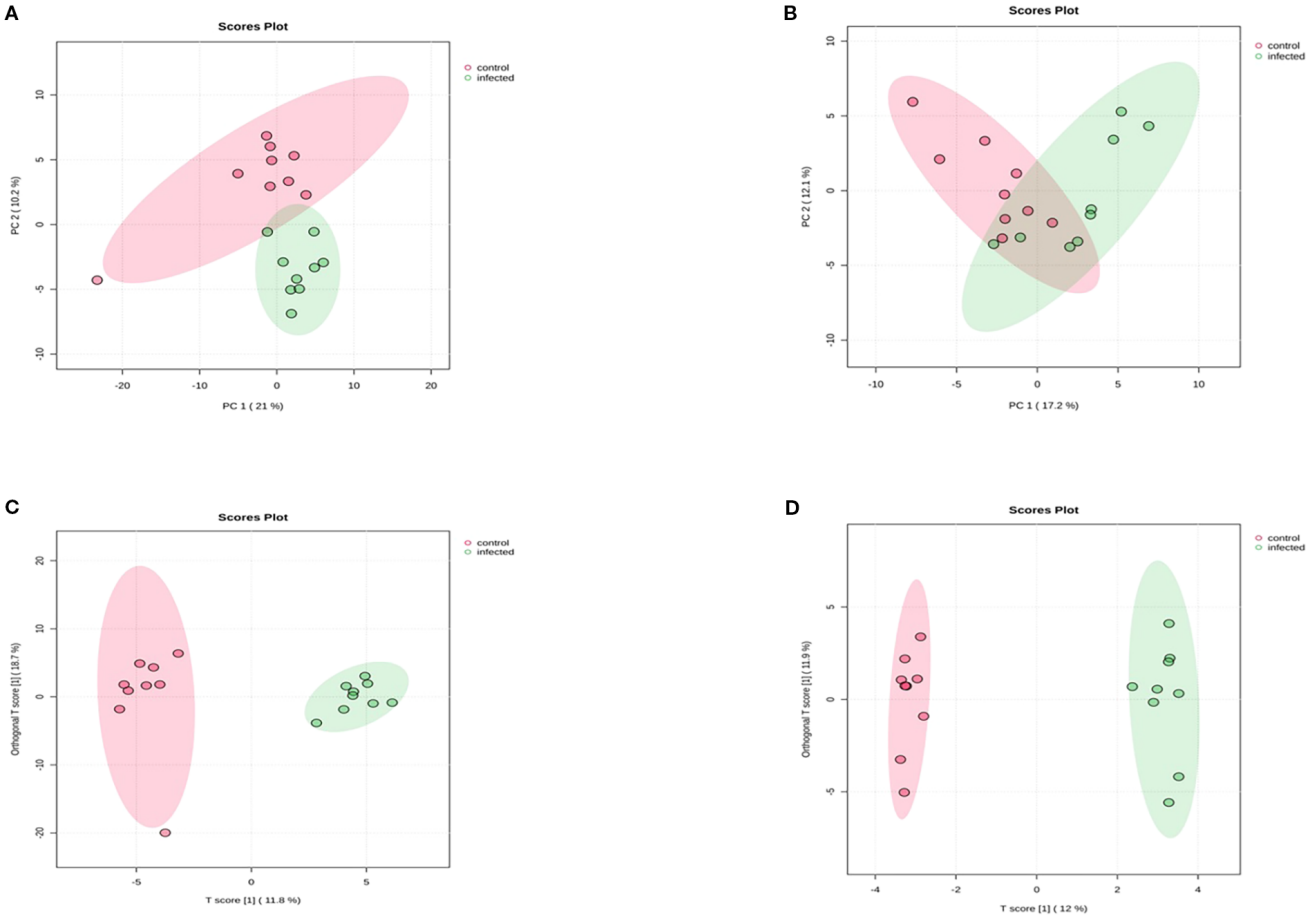
Score Scatter plots of PCA and OPLS-DA of chicken lung metabolomic profiling following HPAI H5N1 infection **(A)** PCA score plot Positive ionization mode **(B)** PCA score plot negative ionization mode **(C)** OPLS-DA score plot positive ionization mode **(D)** OPLS-DA score plot negative ionization mode. Each point in the figure represents a sample, and samples from the same group are represented by the same color.

PCA analysis showed slight overlap between infected and control samples in both ionization modes, as depicted in **Figures 1A, B**. In contrast, OPLS-DA analysis (**Figures 1C, D**) clearly separated infected and control samples in both modes. The OPLS-DA model yielded matrix values of R^2^X = 0.131, R^2^Y = 0.884, and Q^2^ = 0.645 for positive ionization mode, and R^2^X = 0.138, R^2^Y = 0.852, and Q^2^ = 0.646 for negative ionization mode. These Q^2^ values indicate that the experimental models are reliable, reflecting significant alterations in cellular metabolism following HPAI H5N1 infection.

**Figure 2:**
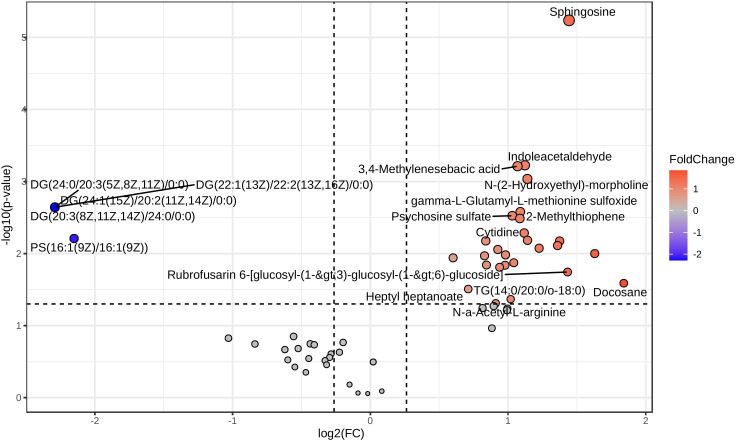
Volcano plot of differential metabolites in chicken lung metabolomic profiling following HPAI H5N1 infection in positive ionization mode. Each point in the plot represents a metabolite: red dots indicate upregulated metabolites, blue dots indicate downregulated metabolites, and gray dots represent metabolites with no significant change.

**Figure 3:**
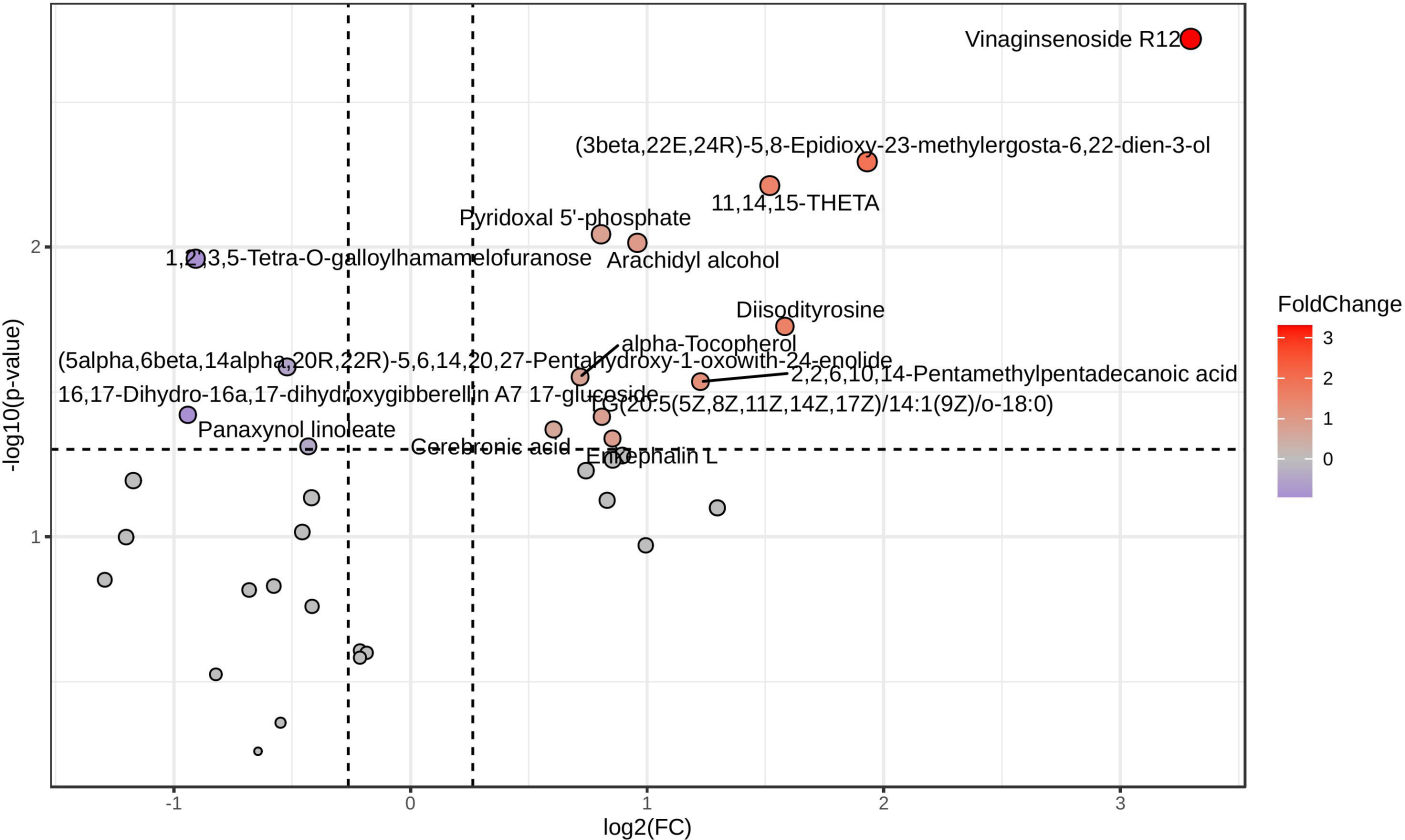
Volcano plot of differential metabolites in chicken lung metabolomic profiling following HPAI H5N1 infection in negative ionization mode. Each point in the plot represents a metabolite: red dots indicate upregulated metabolites, blue dots indicate downregulated metabolites, and gray dots represent metabolites with no significant change.

**Figure 4:**
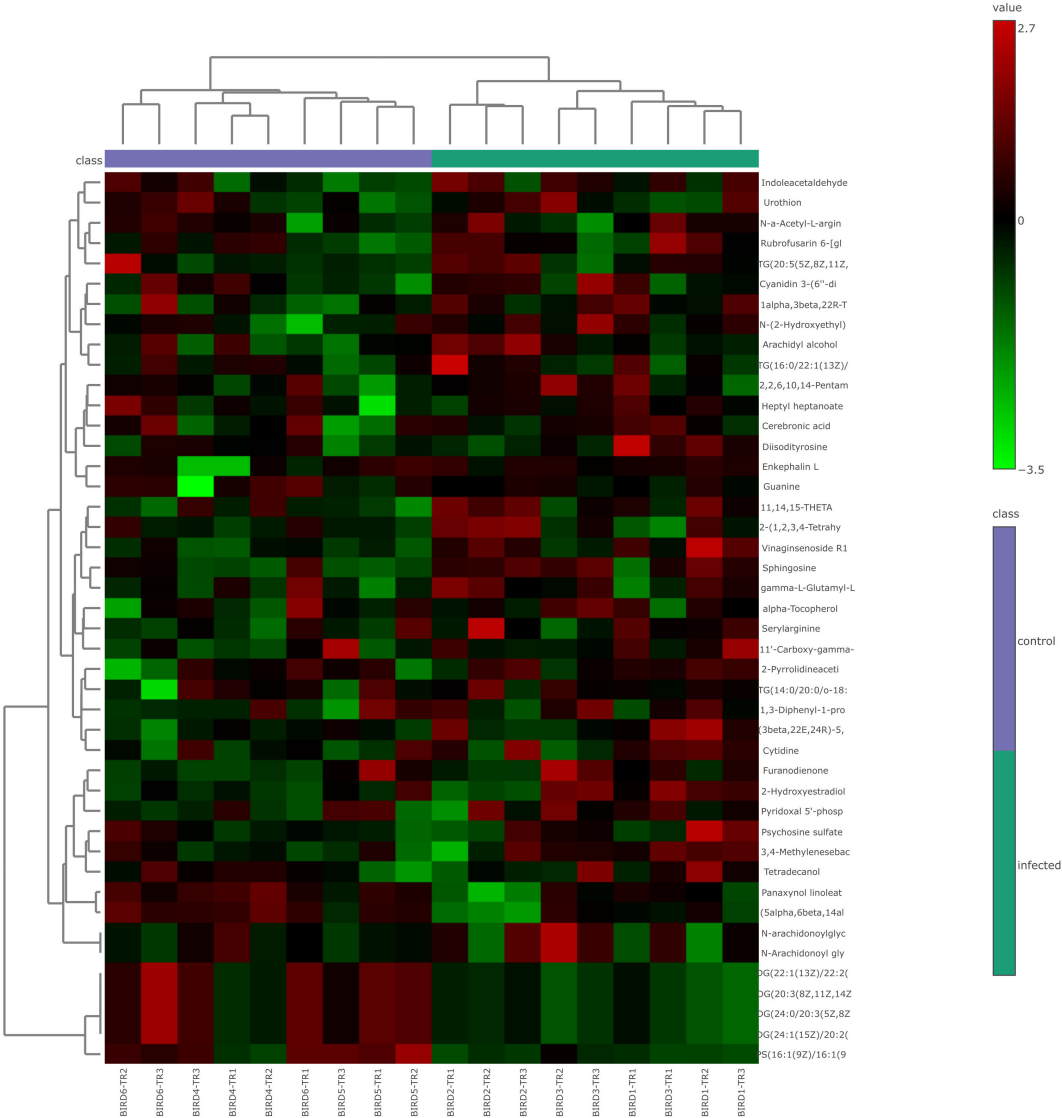
Heat map of combined differential metabolites in positive and negative ionization modes from lung metabolomic profiling following HPAI H5N1 infection in chicken. Each column in the heat map represents an individual sample, and each row represents a differential metabolite. The color of each cell indicates the relative level of the differential metabolites: red signifies upregulation, while green indicates downregulation.

After retaining metabolites with VIP scores ≥ 1, 65 metabolites remained in positive ionization mode and 36 in negative ionization mode. Subsequent t-test and fold change (FC) analysis identified 31 significantly differentially expressed metabolites in positive ionization mode (26 upregulated and 5 downregulated) and 13 in negative ionization mode (11 upregulated and 2 downregulated). Notable metabolites among the significantly differentially expressed ones include psychosine sulfate, sphingosine, indole acetaldehyde, 11,14,15-THETA, diglycerides (DG), and phosphatidylserine (PS). These are represented as volcano plots in **Figure 2** for positive ionization mode and **Figure 3** for negative ionization mode. A heat map of the combined differential metabolites from both positive and negative ionization modes is shown in **Figure 4**. The complete list of significantly differentially expressed metabolites provides in **Supplementary Tables 2**, **3**.

**Figure 5:**
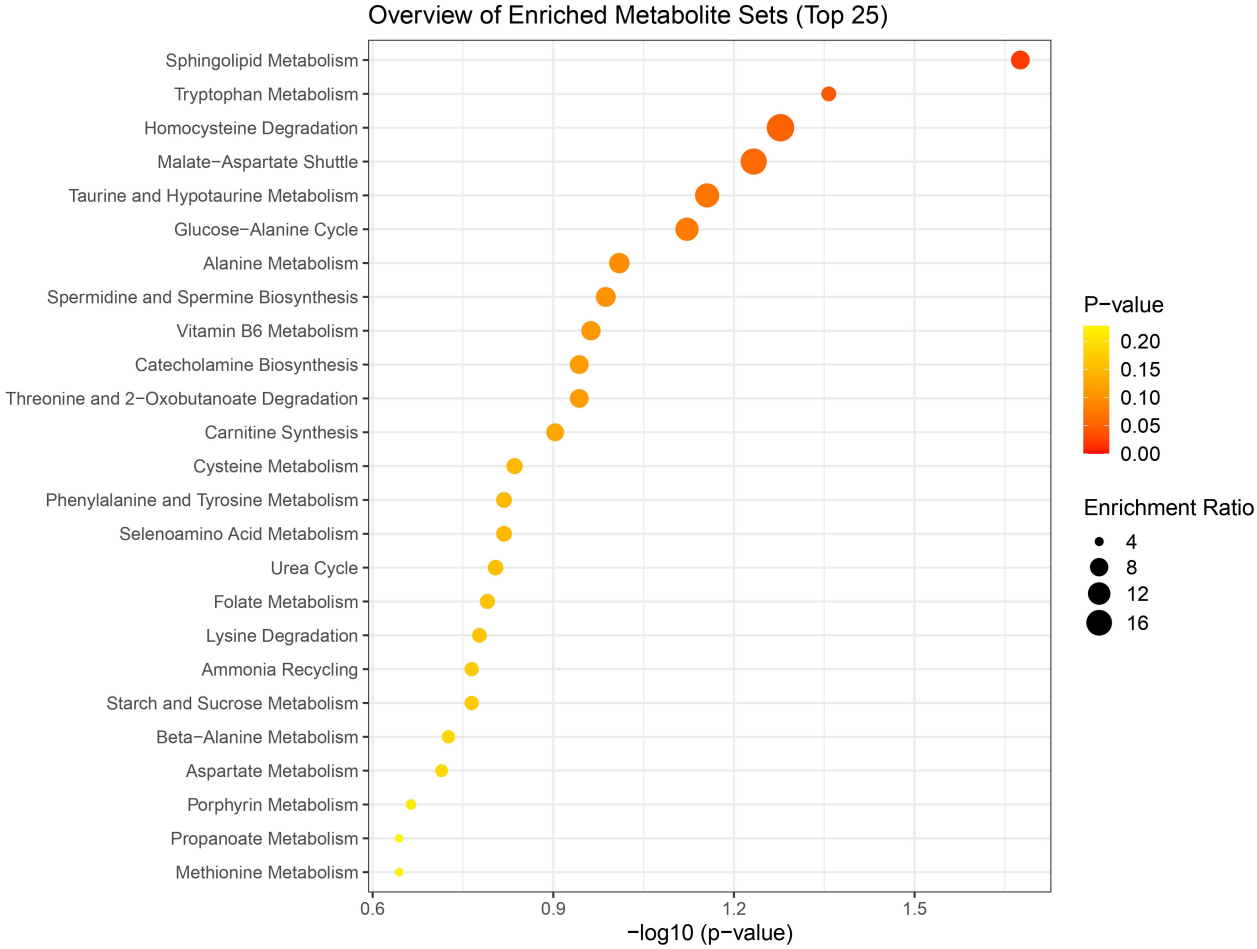
Bubble plot diagram of metabolic pathways enriched in chicken lung tissue following HPAI H5N1 infection. Each bubble represents a metabolic pathway, with the 25 most significant pathways displayed based on their p-values. The size of each bubble corresponds to the number of metabolites involved in the pathway. The x-axis represents the p-value of the pathway, while the y-axis represents the enriched pathways.

Pathway enrichment analysis of the combined list of differential metabolites from both ionization modes (a total of 44 metabolites) identified key metabolic pathways, including Sphingolipid metabolism, Tryptophan metabolism, Homocysteine degradation, and the Malate-Aspartate shuttle. Notably, Sphingolipid and Tryptophan metabolism pathways were significantly enriched with p-values ≤ 0.05. A dot plot of the pathway enrichment analysis is presented in **Figure 5**, and the detailed results are provided in the **Supplementary Table 6**.

### 3.2 Serum metabolomic profiling

LC-MS/MS-based metabolomic profiling of serum samples identified 3,866 aligned peaks in positive ionization mode and 2,660 in negative ionization mode using MZMine 2.53. The MS2Compound search assigned 1,129 metabolites in positive and 545 in negative ionization mode. After excluding exogenous metabolites, 145 metabolites in positive ionization mode and 107 in negative ionization mode were retained.

**Figure 6:**
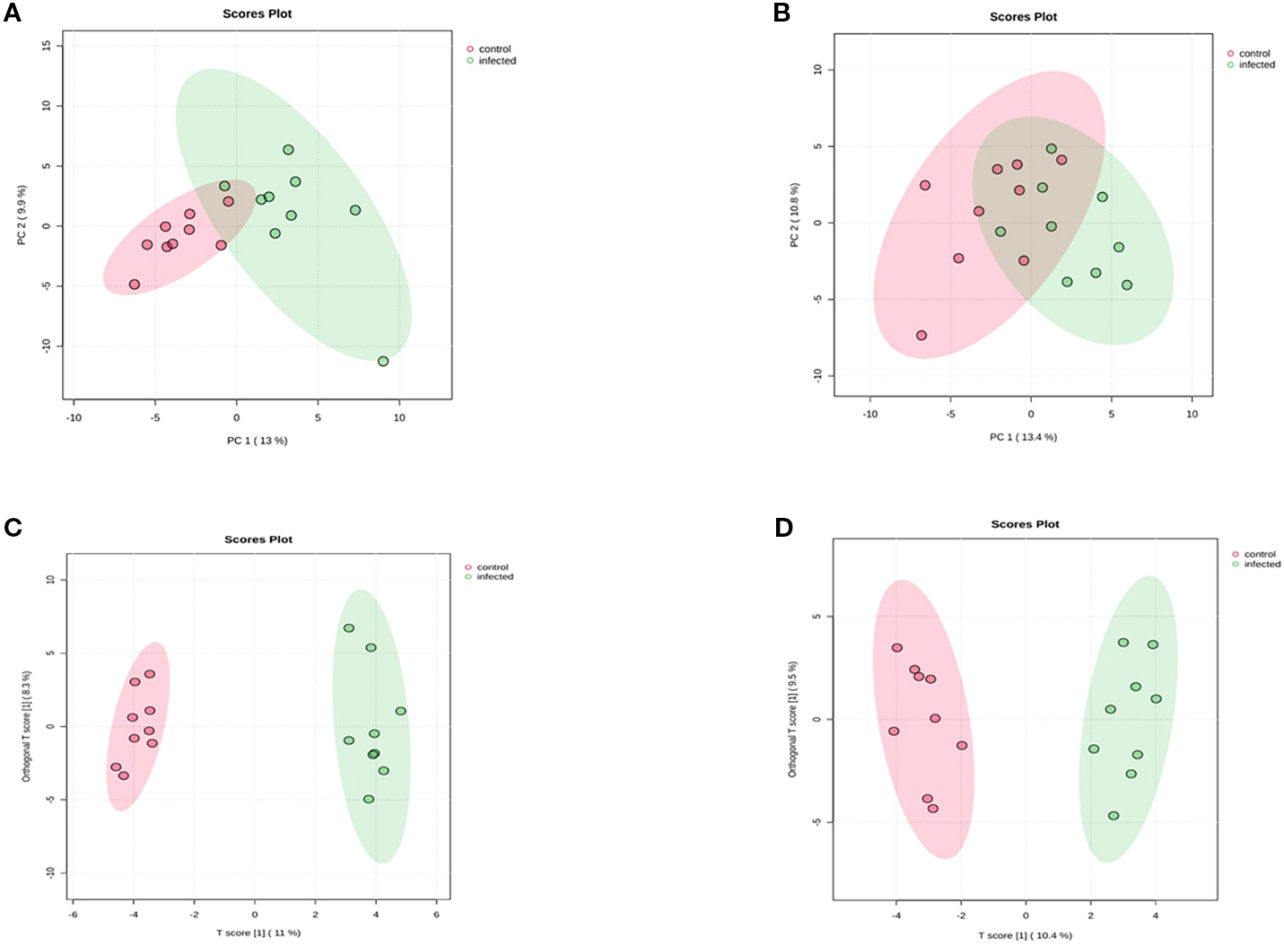
Score Scatter plots of PCA and OPLS-DA of chicken serum metabolomic profiling following HPAI H5N1 infection **(A)** PCA score plot Positive ionization mode **(B)** PCA score plot negative ionization mode **(C)** OPLS-DA score plot positive ionization mode **(D)** OPLS-DA score plot negative ionization mode. Each point in the figures represents a sample, and samples from the same group are represented by the same color.

Similar to the lung sample profiling, PCA analysis of serum samples showed a slight overlap between infected and control samples in both ionization modes (**Figures 6A, B**). However, OPLS-DA analysis (**Figures 6C, D**) revealed a clear separation between infected and control samples. The matrix values obtained after OPLS-DA were R^2^X = 0.11, R^2^Y = 0.906, and Q^2^ = 0.605 for positive ionization mode, and R^2^X = 0.104, R^2^Y = 0.855, and Q^2^ = 0.419 for negative ionization mode. The Q^2^ values in both ionization modes were adequate to consider the models trustworthy.

**Figure 7:**
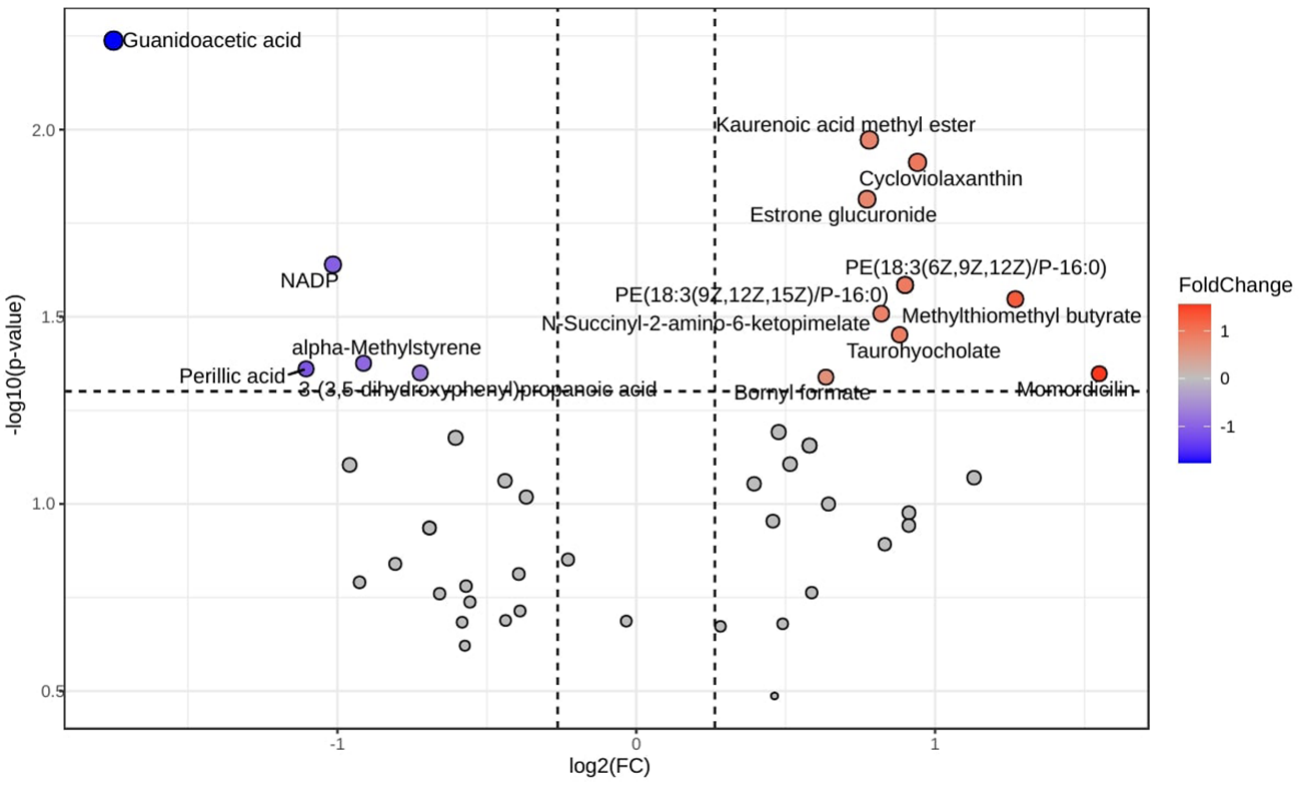
Volcano plot of differential metabolites in chicken serum metabolomic profiling following HPAI H5N1 infection in positive ionization mode. Each point in the volcano map represents a metabolite. Red dots represents upregulation, blue dots represents down regulation and gray dot represents not significant.

**Figure 8:**
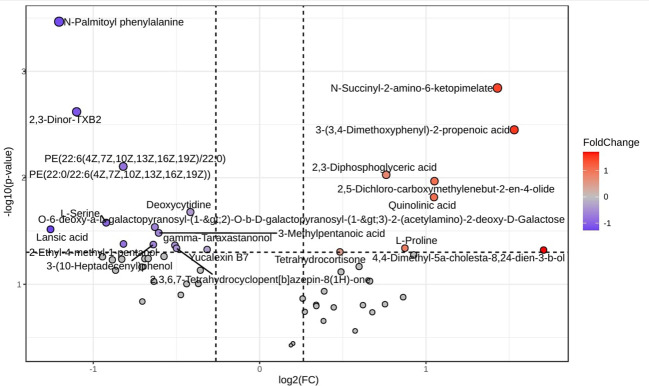
Volcano plot of differential metabolites in chicken serum metabolomic profiling following HPAI H5N1 infection in negative ionization mode. Each point in the volcano map represents a metabolite. Red dots represents upregulation, blue dots represents down regulation and gray dot represents not significant.

**Figure 9:**
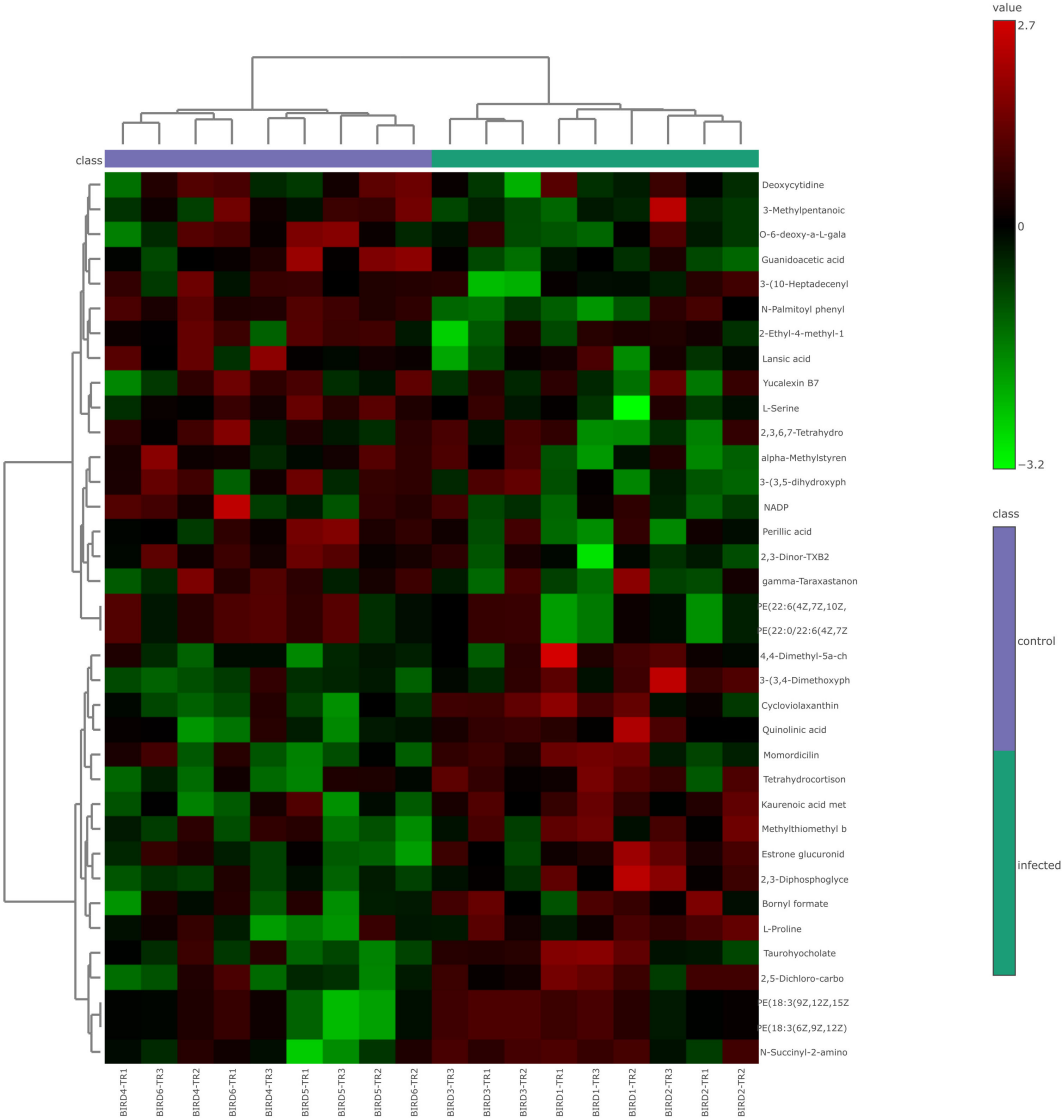
Heat map of combined differential metabolites in positive and negative ionization modes from serum metabolomic profiling following HPAI H5N1 infection in chicken. Each column in the heat map represents an individual sample, and each row represents a differential metabolite. The color of each cell indicates the relative level of the differential metabolites: red signifies upregulation, while green indicates downregulation.

After retaining metabolites with VIP scores >1, 55 metabolites were identified in positive ionization mode and 49 in negative ionization mode. Following t-test and fold change (FC) analysis, 22 significantly differentially expressed metabolites were identified in positive ionization mode, with 8 upregulated and 14 downregulated. In negative ionization mode, 15 differentially expressed metabolites were found, with 10 upregulated and 5 downregulated. The complete list of significantly differentially expressed metabolites provides in **Supplementary Tables 4** and **5** and visualized as a volcano plots (**Figures 7**, **8**). A heat map of the combined differential metabolites from positive and negative ionization modes is shown in **Figure 9**. Notable metabolites include 2,3-Diphosphoglyceric acid, 2,3-Dinor-TXB2, Quinolinic acid, N-Palmitoyl phenylalanine, L-Serine, L-Proline, PE(18:3(6Z,9Z,12Z)/P-16:0), PE(18:3(9Z,12Z,15Z)/P-16:0), N-Succinyl-2-amino-6-ketopimelate, and NADP.

**Figure 10:**
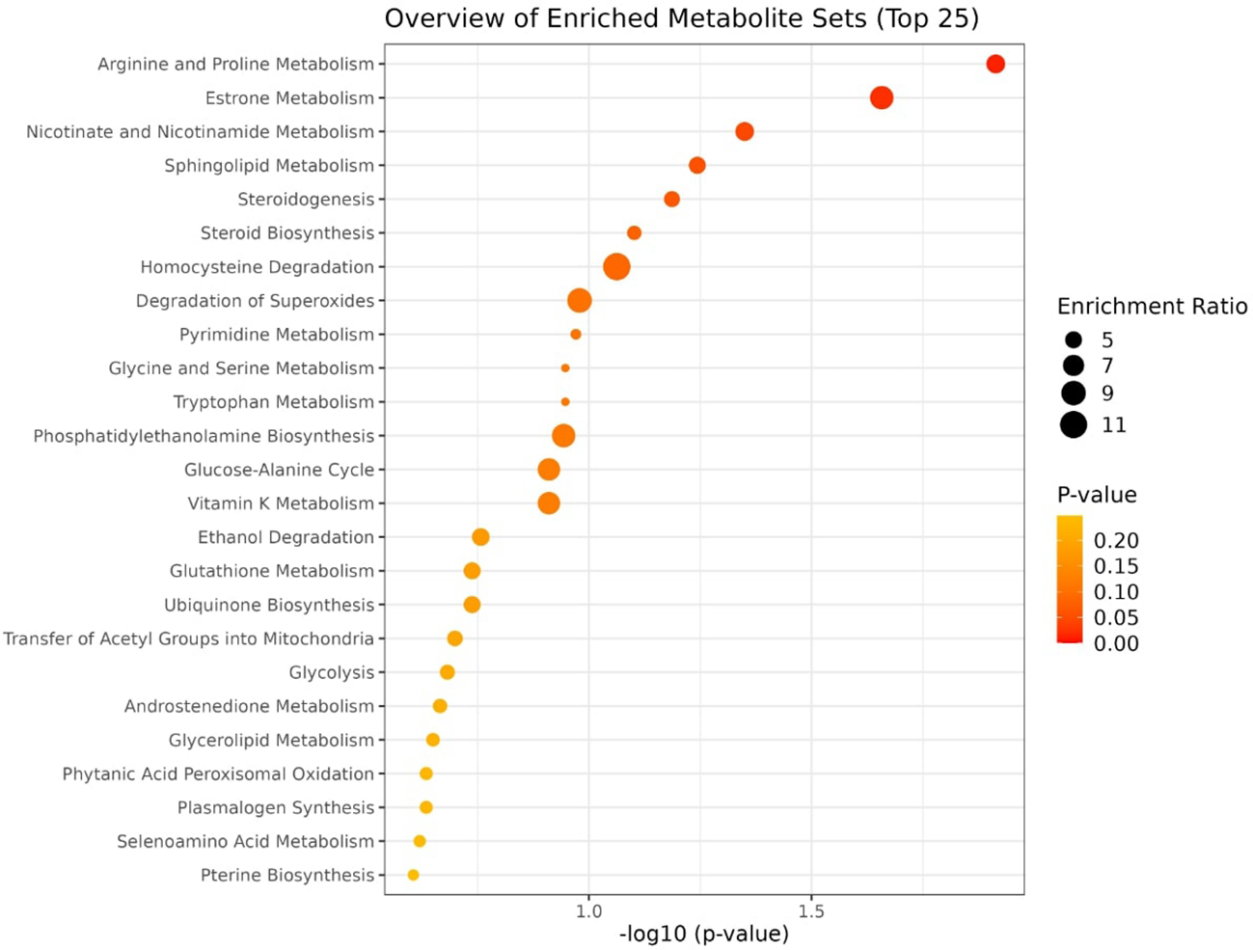
Bubble plot diagram of metabolic pathways enriched in the serum of chicken following HPAI H5N1 infection. Each bubble represents a metabolic pathway, with the 25 most significant pathways displayed based on their p-values. The size of each bubble corresponds to the number of metabolites involved in the pathway. The x-axis represents the p-value of the pathway, while the y-axis represents the enriched pathways.

Pathway enrichment analysis of the combined list of differentially expressed metabolites (a total of 37) identified significant enrichment in Arginine and Proline Metabolism, Estrone Metabolism, Nicotinate and Nicotinamide Metabolism, Sphingolipid Metabolism, and Tryptophan Metabolism. Of these, Arginine and Proline Metabolism, Estrone Metabolism, and Nicotinate and Nicotinamide Metabolism were significantly enriched with a p-value ≤ 0.05. A dot plot of the pathway enrichment analysis is presented in **Figure 10**, and detailed results are provided in **Supplementary Table 7**.

### 3.3 Marker identification

#### 3.3.1 Metabolomic marker identification of lung sample

**Figure 11:**
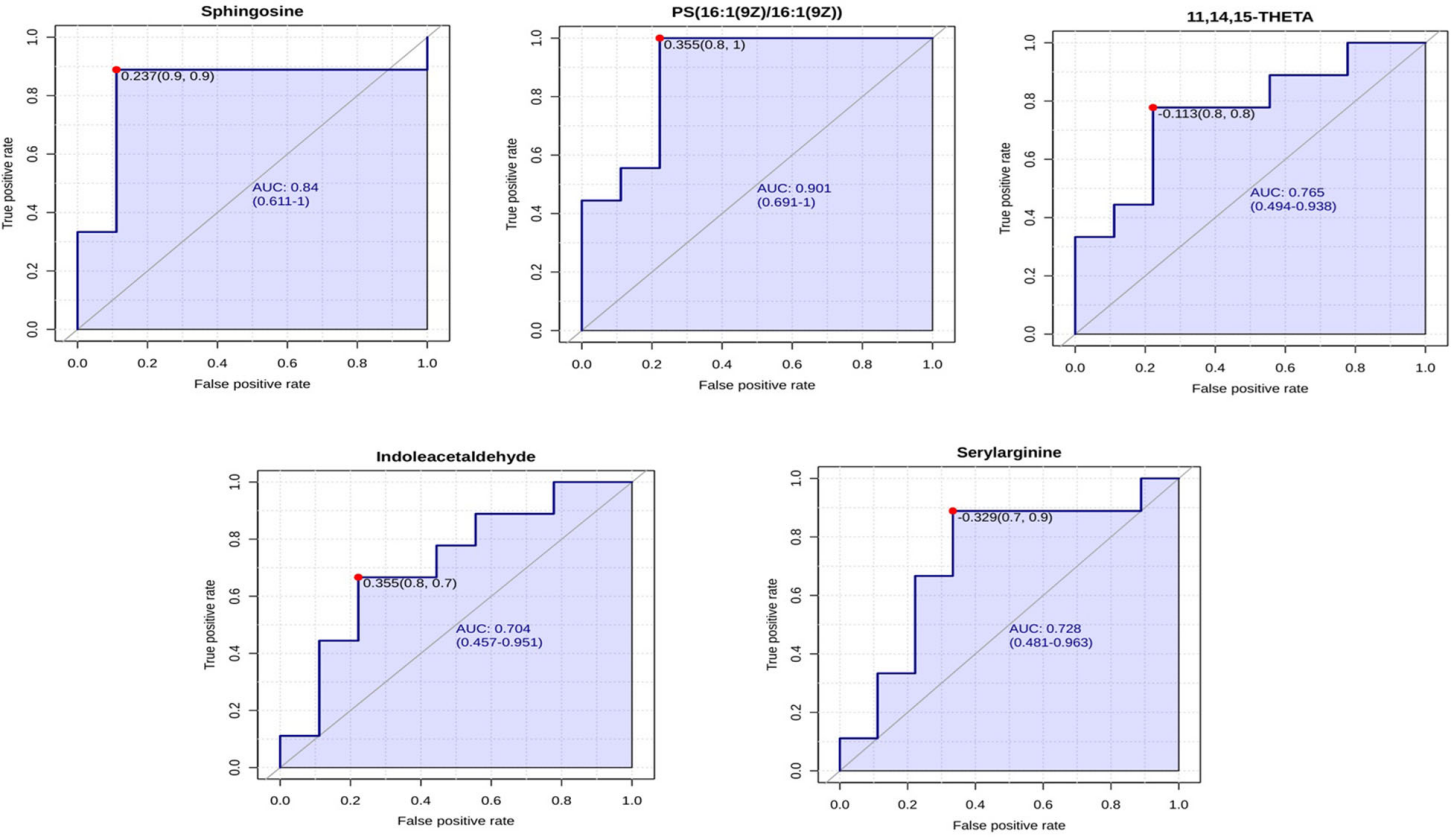
ROC curve for biomarkers identified in the metabolomic profiling of chicken lungs following HPAI H5N1 infection. The X-axis represents the false positive rate, while the Y-axis represents the true positive rate.

ROC curve analysis was conducted on significantly differential metabolites identified in lung samples. AUC values for all the differential metabolites identified metabolites are given in **Supplementary Table 8**. Metabolites with AUC values greater than 0.7 were selected, resulting in 25 metabolites meeting this criterion. Based on their roles in major pathways and existing data linking them to influenza A virus pathogenesis in chicken, potential metabolomic markers identified include Sphingosine (AUC = 0.84), PS(16:1(9Z)/16:1(9Z)) (AUC = 0.901), 11,14,15-THETA (AUC = 0.765), and Indoleacetaldehyde (AUC = 0.704). The ROC plots of these metabolites are shown in **Figure 11**.

#### 3.3.2 Metabolomic marker identification of serum sample

**Figure 12:**
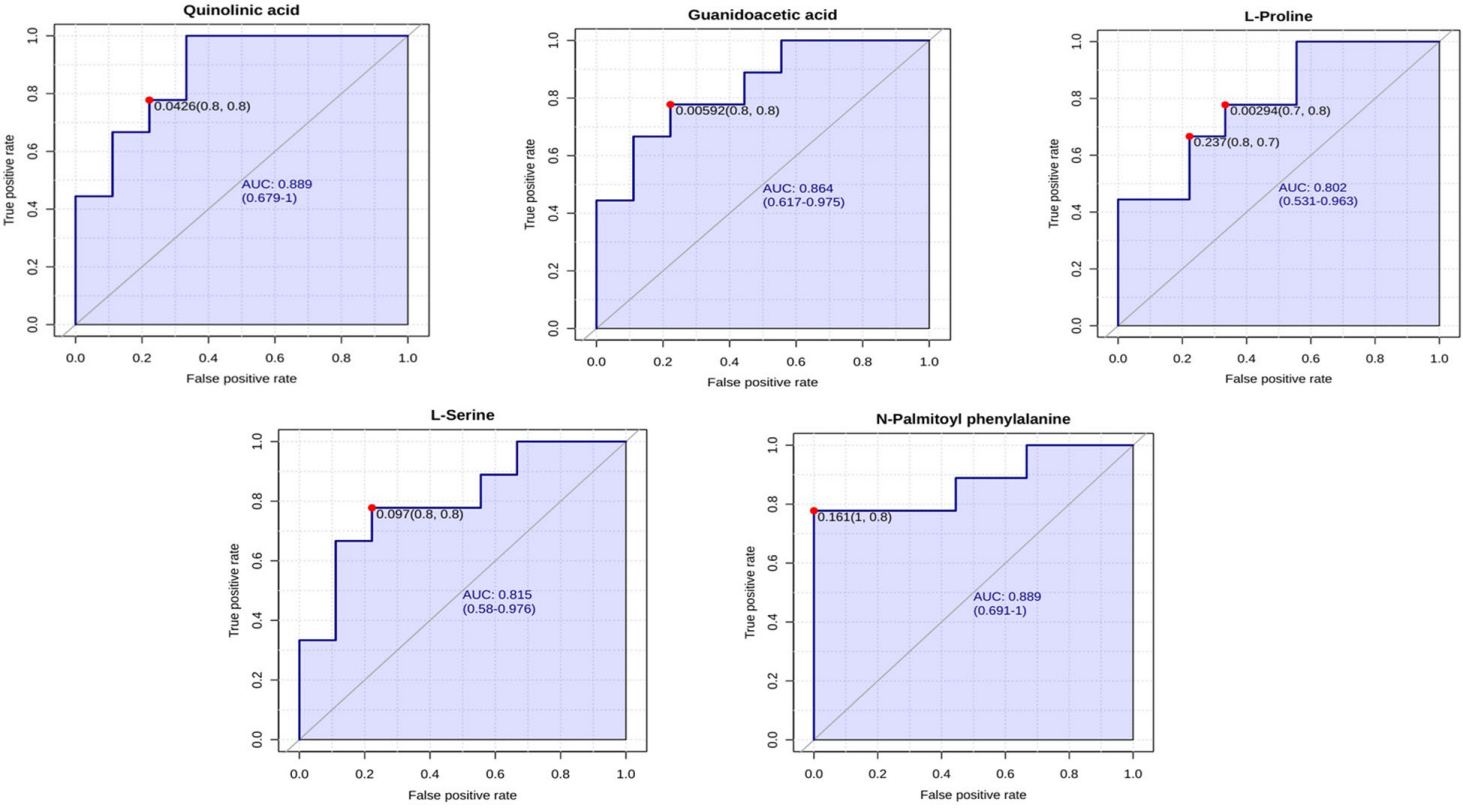
ROC curve for biomarkers identified in the serum metabolomic profiling of chickens following HPAI H5N1 infection. The X-axis represents the false positive rate, while the Y-axis represents the true positive rate.

Similar to the lungs, 31 metabolites in the serum samples showed AUC values greater than 0.7. The AUC values for all differential metabolites in serum are given in **Supplementary Table 9**. Quinolinic acid (AUC = 0.889), Guanidoacetic acid (AUC = 0.864), L-Proline (AUC = 0.802), L-Serine (AUC = 0.815), and N-Palmitoyl phenylalanine (AUC = 0.889) are proposed as potential metabolomic markers in serum following HPAI H5N1 infection. The ROC curve plots for these metabolites are shown in **Figure 12**.

## 4 Discussion

In recent years, metabolomics has significantly advanced our understanding of host-pathogen interactions across various pathogens. As the latest addition to the omics toolkit, metabolomics offers distinct advantages over genomics, transcriptomics, and proteomics. In chicken, metabolomic profiling has been applied to several viral diseases, including Newcastle disease, Marek’s disease, Avian leukosis, and Infectious Bursal Disease (IBD). In this study, we analyzed the metabolomics profile of Highly Pathogenic Avian Influenza (H5N1) infection in chicken using liquid chromatography coupled with tandem mass spectrometry (LC-MS/MS) on a QTRAP 6500 mass spectrometer (AB Sciex) paired with an Agilent 1290 Infinity II liquid chromatography system. This research provides new insights into the chicken host’s response to H5N1 infection and sheds light on virus-host interactions, potentially elucidating the infection mechanisms of HPAI H5N1.

Significant metabolite changes observed in this study were primarily related to lipid metabolism. In the lungs, 15 metabolites in the positive ionization mode and 11 in the negative ionization mode were associated with lipid metabolism. Similarly, in serum, 10 metabolites were linked to lipid metabolism in both positive and negative ionization modes. Changes in lipid metabolism following influenza A virus infection have been reported in multiple studies (Chen et al., 2023; Petrich and Chiantia, 2023; Kawabata et al., 2023). In our study, triglycerides TG (16:0/22:1(13Z)/o-18:0) and TG (14:0/20:0/o-18:0) were upregulated, consistent with findings by Jonkers et al. (2002), who demonstrated hypertriglyceridemia following inflammation. This hypertriglyceridemia can be linked to the inflammation caused by HPAI H5N1 infection.

In the lungs, sphingosine and psychosine sulfate were found to be upregulated in infected samples. Sphingosine is a key component of sphingolipid metabolism, while psychosine sulfate is a glycosphingolipid and a sulfated form of psychosine, according to PubChem. Psychosine is derived from sphingosine through direct galactosylation (Igisu and Suzuki, 1984). The observed upregulation of sphingosine and psychosine sulfate suggests a significant alteration in sphingolipid metabolism following Highly Pathogenic Avian Influenza (H5N1) virus infection. Sphingolipid metabolism is crucial in viral pathogenesis, as sphingolipids facilitate essential structural interactions, including the fusion of the plasma membrane with the viral membrane, which supports viral endocytosis, cell signaling, and viral budding (Avota et al., 2021). These findings indicate that the HPAI H5N1 virus primarily exploit the sphingolipid metabolic pathway for its entry and replication. Alteration of sphingolipid metabolism has been reported by Zhang et al. (2024b) following experimental infection of H9N2 avian influenza virus in chick DF1 cells.

Similarly, we identified that indole acetaldehyde and pyridoxal 5’-phosphate are upregulated in infected lung tissue, while quinolinic acid is upregulated in infected serum. These metabolites are part of tryptophan metabolism according to Human Metabolomic Database (HMDB). Quinolinic acid is specifically involved in the kynurenine pathway of tryptophan metabolism and pyridoxal 5’-phosphate serves as a coenzyme for several enzymes in the tryptophan metabolism, particularly in the kynurenine pathway (Stone and Darlington, 2002; Rios-Avila et al., 2013; Stone, 2016). The differential expression of these metabolites suggests a significant link between HPAI H5N1 infection and tryptophan metabolism, particularly the kynurenine pathway. The association between influenza A virus and tryptophan metabolism has been established by several studies (Cui et al., 2016; Chandler et al., 2016; Gaelings et al., 2017). Alterations in tryptophan metabolism are linked to inflammation and the metabolites within the kynurenine pathway contribute to immunomodulation and changes in central nervous system function (Chandler et al., 2016; Gaelings et al., 2017). Therefore, we can conclude that HPAI H5N1 leverages tryptophan metabolism to induce lung injury and inflammation.

The metabolite 11,14,15-THETA is upregulated in the lungs following infection. It is a product of the 15-lipoxygenase (15-LO) pathway of arachidonic acid, as reported by Pfister et al., 1998. A study by Zhu et al. (2003) also identified an increased production of 15-LO pathway products, including 15-hydroxyeicosatetraenoic acid (HETE), 11,14,15-trihydroxyeicosatrienoic acid (THETA), and 11,12,15-THETA, in response to chronic hypoxia in neonatal rabbits. The 15-LO pathway is implicated in hypoxic pulmonary vasoconstriction (HPV), a homeostatic mechanism intrinsic to the pulmonary vasculature (Zhu and Ran, 2012). HPV involves the constriction of intrapulmonary arteries in response to alveolar hypoxia and diverting blood to better oxygenated lung segments, thereby optimizing ventilation/perfusion matching and systemic oxygen delivery (Dunham-Snary et al., 2017). The elevation of 11,14,15-THETA production could thus be linked to hypoxic conditions caused by lung pathology induced by the HPAI H5N1 virus.

In serum profiling, we detected elevated levels of 2,3-Diphosphoglyceride (2,3-DPG or 2,3-BPG) in infected birds. 2,3-DPG is an intermediate metabolite in the Luebering–Rapoport glycolytic pathway, synthesized in red blood cells (RBCs) from 1,3-diphosphoglycerate (1,3-DPG) through the action of diphosphoglycerate mutase. It functions as a regulator of hemoglobin’s allosteric properties in RBCs. When 2,3-DPG binds to hemoglobin, it stabilizes the T-state conformation, thereby reducing hemoglobin’s affinity for oxygen and helps in release of oxygen (Benesch and Benesch, 1967; Brewer, 1974). Production of 2,3-DPG increases in response to hypoxia. Acute hypoxic exposure induces hyperventilation, enhances CO2 removal, and leads to respiratory alkalosis, which raises blood pH (Lühker et al., 2017). This elevated pH stimulates glycolysis, contributing to an increased concentration of 2,3-DPG (Duhm and Gerlach, 1971). Although 2,3-DPG’s role as an allosteric regulator is more pronounced in mammals, it has also been observed in avian species (Isaacks et al., 1977). Therefore, the increased level of 2,3-DPG may be associated with hypoxia resulting from lung inflammation due to infection.

Our study also identified several amino acids with significant changes between infected and control samples. Notable alterations include serine, proline, seryl arginine, and N-a-Acetyl-L-arginine. Seryl arginine and N-a-Acetyl-L-arginine are derivatives of arginine. Arginine metabolism is essential for influenza virus replication, with studies showing reduced virus yield in arginine-depleted cultures (Schierhorn et al., 2017; Becht, 1969). Additionally, serine, involved in sphingolipid metabolism, is crucial for various stages of influenza virus lifecycle, including entry, budding, and propagation (Avota et al., 2021). Thus, alterations in amino acid metabolism likely play a significant role in viral propagation within the host. It may be noted that the metabolite level changes observed in the lungs and serum are indicative of the initial inflammation and host responses to HPAI H5N1infection, however, clinically only slight dullness was observed in the infected birds at 12hr post inoculation. This might be due to the fact that though the metabolite changes have just started to appear in the lungs and serum, the clinical impacts were so far only minimal owing to homeostatic mechanisms active in other tissues in intact birds.

By comparing the pathway profiles of the lungs and serum after infection, we identified several commonly affected pathways, including sphingolipid metabolism, tryptophan metabolism, homocysteine degradation, and the glucose-alanine cycle. Additionally, there are pathways uniquely impacted in the lungs, such as the malate-aspartate shuttle, taurine and hypotaurine metabolism, and spermidine and spermine metabolism. In contrast, the serum exhibited alterations in pathways related to arginine and proline metabolism, estrone metabolism, and nicotinate and nicotinamide metabolism, among others.

Pathways such as homocysteine degradation, estrone metabolism, taurine and hypotaurine metabolism, nicotinate and nicotinamide metabolism, and steroidogenesis show high enrichment in our study following infection. However, the roles of these pathways are not well characterized with respect to influenza, especially in mammals. Therefore, these pathways could be uniquely related to avian species, but further research is needed to understand their actual roles in chicken following infection.

We have identified potential metabolomic biomarkers associated with HPAI H5N1 infection in chicken. Through ROC curve analysis, metabolites with an AUC value exceeding 0.7 were deemed as potential biomarker candidates. From these candidates, we selected specific metabolomic markers based on their roles in metabolic pathways that exhibited significant enrichment (p < 0.05) and had prior studies linking them to the influenza A virus. We propose sphingosine, PS(16:1(9Z)/16:1(9Z)), 11,14,15-THETA, and indoleacetaldehyde as metabolomic markers in the lungs following HPAI H5N1 infection. Sphingosine is involved in the sphingolipid metabolic pathway, which showed significant enrichment (p < 0.05) in our study. Similarly, indoleacetaldehyde is part of the tryptophan metabolism pathway, which also demonstrated significant enrichment (p < 0.05). PS(16:1(9Z)/16:1(9Z)) was chosen due to its established association with influenza A virus infection, as noted by Shiratsuchi et al. (2000) and Moller-Tank and Maury (2014). Additionally, 11,14,15-THETA, a product of the 15-lipoxygenase pathway of arachidonic acid, has been linked to hypoxia, as reported by Zhu et al. (2003). Given that hypoxia can arise from lung injury caused by the influenza virus, 11,14,15-THETA is considered a relevant biomarker. For serum analysis, we identified quinolinic acid, guanidoacetic acid, L-proline, L-serine, and N-palmitoyl phenylalanine as potential biomarkers. Quinolinic acid is part of the tryptophan metabolism pathway, which has been associated with influenza in multiple studies (Cui et al., 2016; Chandler et al., 2016; Gaelings et al., 2017). Guanidoacetic acid is linked to the arginine and proline metabolism pathway, which is significantly enriched, with its correlation to the influenza A virus documented by Schierhorn et al. (2017) and Becht (1969). L-proline and L-serine, both amino acids, along with N-palmitoyl phenylalanine, a phenylalanine derivative, are vital for viral development, making them strong candidates for metabolomic markers.Further validation is necessary to confirm these suggested metabolites through testing with field samples of a larger size and targeted metabolome analysis. Other metabolites with strong AUC values may also serve as markers, but currently, there is insufficient data linking them directly to HPAI H5N1 infection.

## 5 Conclusion

Our study identified significant alterations in metabolites and pathways following HPAI H5N1 infection with more pronounced alterations in the lungs compared to serum suggesting lungs is the primary site of infection. We found notable enrichment in sphingolipid metabolism, tryptophan metabolism, and arginine and proline metabolism in chicken, aligning with findings in mammals. The alterations in the sphingolipid pathway suggest that the virus may utilize it for structural interactions, while changes in tryptophan metabolism could explain the central nervous system (CNS) signs often observed after infection. Due to the absence of a comprehensive chicken-specific metabolome database, we relied on human databases for metabolite and pathway identification. Nonetheless, our findings will contribute to the development of a dedicated chicken metabolome database and support future research. The biomarkers identified in this study may serve as potential tools for disease diagnosis following proper validation. Further investigation into these metabolic changes can enhance our understanding of HPAI pathogenesis and facilitate advancements in disease diagnosis and control.

## Data Availability

The original contributions presented in the study are publicly available. This data can be found here: http://massive.ucsd.edu/ProteoSAFe/status.jsp?task=3afef197172545bdb49069838848e704 with access number/study ID MSV000097417.

## Supplementary material

Supplementary Material 1 Table 1-9.

Supplementary Material 2 Comprehensive metabolomic profiling data of lung and serum samples following HPAI H5N1 infection in chicken in both Positive and negative ionization modes.
