# Supplementary material for: Metabolomic profiling and identification of potential biomarkers of highly pathogenic avian influenza (H5N1) in chicken
Source: Front Cell Infect Microbiol. 2025 Oct 7;15:1540290. doi: 10.3389/fcimb.2025.1540290 (PMC12537707; doi:10.3389/fcimb.2025.1540290)
Supplement: Supplementary file 2 [file Table1.docx]

**Table 1- Gradient applied for Liquid Chromatography**

| **Time (mins)** | **% Solvent A** | **% Solvent B** |
| --- | --- | --- |
| 1 | 98 | 2 |
| 2 | 80 | 20 |
| 7 | 75 | 25 |
| 8 | 70 | 30 |
| 9 | 65 | 35 |
| 10 | 60 | 40 |
| 11 | 55 | 45 |
| 12 | 50 | 50 |
| 14 | 45 | 55 |
| 15 | 40 | 60 |
| 18 | 5 | 95 |
| 19 | 5 | 95 |
| 21 | 98 | 2 |
| 25 | 98 | 2 |

**Solvent A** - 0.1% formic acid in LC-MS grade water

**Solvent B** - 0.1% formic acid in 90% acetonitrile.

**The flow rate** - 0.250 mL/min.

| \| **No** \| **Name** \| **HMDB** \| **row m/z** \| **row retention time** \| **VIP** \| **Fold Change** \| **p-value** \| \| --- \| --- \| --- \| --- \| --- \| --- \| --- \| --- \| \| 1 \| 1alpha,3beta,22R-Trihydroxyergosta-5,24E-dien-26-oic acid 3-O-b-D-glucoside 26-O-b-D-glucosyl ester \| HMDB0040398 \| 785.4 \| 19.95105 \| 1.646774 \| 2.5665 \| 0.007738 \| \| 2 \| 2-Hydroxyestradiol \| HMDB0000338 \| 289.2 \| 5.077098 \| 1.422264 \| 3.0945 \| 0.010009 \| \| 3 \| gamma-L-Glutamyl-L-methionine sulfoxide \| HMDB0036170 \| 205.2 \| 4.424826 \| 1.540048 \| 2.1276 \| 0.002633 \| \| 4 \| 3,4-Methylenesebacic acid \| HMDB0059729 \| 227.16 \| 9.224764 \| 1.705376 \| 2.0982 \| 0.000613 \| \| 5 \| Cyanidin 3-(6''-dioxalylglucoside) \| HMDB0039936 \| 594.12 \| 18.89139 \| 1.439776 \| 1.9 \| 0.008777 \| \| 6 \| 1,3-Diphenyl-1-propanone \| HMDB0032041 \| 211.08 \| 10.66329 \| 1.279889 \| 1.7944 \| 0.014413 \| \| 7 \| Heptyl heptanoate \| HMDB0034461 \| 229.2 \| 2.099057 \| 1.157255 \| 1.6372 \| 0.03107 \| \| 8 \| Psychosine sulfate \| HMDB0013046 \| 542.28 \| 2.936568 \| 1.469278 \| 2.0437 \| 0.002985 \| \| 9 \| Rubrofusarin 6-[glucosyl-(1-&gt;3)-glucosyl-(1-&gt;6)-glucoside] \| HMDB0034569 \| 759.24 \| 20.68531 \| 1.518842 \| 2.6993 \| 0.018026 \| \| 10 \| N-Arachidonoyl glycine \| HMDB0005096 \| 362.28 \| 3.480908 \| 1.116618 \| 1.9751 \| 0.010431 \| \| 11 \| N-arachidonoylglycinate \| HMDB0062617 \| 362.28 \| 3.480908 \| 1.116618 \| 1.9751 \| 0.010431 \| \| 12 \| N-a-Acetyl-L-arginine \| HMDB0004620 \| 217.08 \| 0.87 \| 1.240355 \| 1.8804 \| 0.048812 \| \| 13 \| 11'-Carboxy-gamma-tocotrienol \| HMDB0012518 \| 401.28 \| 18.81434 \| 1.477809 \| 1.9174 \| 0.015485 \| \| 14 \| Tetradecanol \| HMDB0011638 \| 215.28 \| 2.060792 \| 1.456505 \| 1.7882 \| 0.00667 \| \| 15 \| Guanine \| HMDB0000132 \| 152.04 \| 3.652022 \| 1.211688 \| 1.5171 \| 0.011464 \| \| 16 \| TG(16:0/22:1(13Z)/o-18:0) \| HMDB0044220 \| 903.84 \| 19.88725 \| 1.432319 \| 1.9705 \| 0.014523 \| \| 17 \| Sphingosine \| HMDB0000252 \| 300.24 \| 4.431426 \| 2.031566 \| 2.7192 \| 5.81E-06 \| \| 18 \| N-(2-Hydroxyethyl)-morpholine \| HMDB0012262 \| 132.12 \| 4.407676 \| 1.805184 \| 2.2048 \| 0.000921 \| \| 19 \| Furanodienone \| HMDB0012282 \| 231.12 \| 4.328969 \| 1.090656 \| 2.0591 \| 0.013403 \| \| 20 \| 2-(1,2,3,4-Tetrahydroxybutyl)-6-(2,3,4-trihydroxybutyl)pyrazine \| HMDB0012299 \| 305.16 \| 18.8721 \| 1.255027 \| 2.3391 \| 0.008429 \| \| 21 \| Serylarginine \| HMDB0012304 \| 262.2 \| 5.075737 \| 1.504894 \| 2.5932 \| 0.0067 \| \| 22 \| TG(14:0/20:0/o-18:0) \| HMDB0012306 \| 849.84 \| 4.641537 \| 1.184319 \| 2.0271 \| 0.042939 \| \| 23 \| DG(20:3(8Z,11Z,14Z)/24:0/0:0) \| HMDB0012324 \| 731.64 \| 20.13585 \| 2.201796 \| 0.20453 \| 0.002274 \| \| 24 \| DG(22:1(13Z)/22:2(13Z,16Z)/0:0) \| HMDB0012325 \| 731.64 \| 20.13585 \| 2.201796 \| 0.20453 \| 0.002274 \| \| 25 \| DG(24:0/20:3(5Z,8Z,11Z)/0:0) \| HMDB0012326 \| 731.64 \| 20.13585 \| 2.201796 \| 0.20453 \| 0.002274 \| \| 26 \| DG(24:1(15Z)/20:2(11Z,14Z)/0:0) \| HMDB0012327 \| 731.64 \| 20.13585 \| 2.201796 \| 0.20453 \| 0.002274 \| \| 27 \| 2-Pyrrolidineacetic acid \| HMDB0012341 \| 130.08 \| 1.831891 \| 1.555855 \| 2.2063 \| 0.006546 \| \| 28 \| Cytidine \| HMDB0012355 \| 244.08 \| 10.16292 \| 1.617662 \| 2.1698 \| 0.005179 \| \| 29 \| PS(16:1(9Z)/16:1(9Z)) \| HMDB0012361 \| 732.48 \| 20.58729 \| 2.101066 \| 0.22529 \| 0.006179 \| \| 30 \| Urothion \| HMDB0012367 \| 326.04 \| 12.2926 \| 1.266377 \| 1.777 \| 0.010742 \| \| 31 \| Indoleacetaldehyde \| HMDB0012379 \| 160.08 \| 3.82873 \| 1.919339 \| 2.1771 \| 0.000596 \|   **Table 2: List of differential metabolites in positive ionisation mode of lung metabolome profiling following H5N1 infection.** |
| --- | --- | --- | --- | --- | --- | --- | --- | --- | --- | --- | --- | --- | --- | --- | --- | --- | --- | --- | --- | --- | --- | --- | --- | --- | --- | --- | --- | --- | --- | --- | --- | --- | --- | --- | --- | --- | --- | --- | --- | --- | --- | --- | --- | --- | --- | --- | --- | --- | --- | --- | --- | --- | --- | --- | --- | --- | --- | --- | --- | --- | --- | --- | --- | --- | --- | --- | --- | --- | --- | --- | --- | --- | --- | --- | --- | --- | --- | --- | --- | --- | --- | --- | --- | --- | --- | --- | --- | --- | --- | --- | --- | --- | --- | --- | --- | --- | --- | --- | --- | --- | --- | --- | --- | --- | --- | --- | --- | --- | --- | --- | --- | --- | --- | --- | --- | --- | --- | --- | --- | --- | --- | --- | --- | --- | --- | --- | --- | --- | --- | --- | --- | --- | --- | --- | --- | --- | --- | --- | --- | --- | --- | --- | --- | --- | --- | --- | --- | --- | --- | --- | --- | --- | --- | --- | --- | --- | --- | --- | --- | --- | --- | --- | --- | --- | --- | --- | --- | --- | --- | --- | --- | --- | --- | --- | --- | --- | --- | --- | --- | --- | --- | --- | --- | --- | --- | --- | --- | --- | --- | --- | --- | --- | --- | --- | --- | --- | --- | --- | --- | --- | --- | --- | --- | --- | --- | --- | --- | --- | --- | --- | --- | --- | --- | --- | --- | --- | --- | --- | --- | --- | --- | --- | --- | --- | --- | --- | --- | --- | --- | --- | --- | --- | --- | --- | --- | --- | --- | --- | --- | --- | --- | --- | --- | --- | --- | --- | --- | --- | --- | --- | --- | --- | --- | --- | --- | --- |

**Table 3: List of differential metabolites in negative ionisation mode of lung metabolome profiling following H5N1 infection.**

| \| **No** \| **Name** \| **MonoMass** \| **HMDB** \| **row m/z** \| **retention time** \| **VIP** \| **Fold Change** \| **p-value** \| \| --- \| --- \| --- \| --- \| --- \| --- \| --- \| --- \| --- \| \| 1 \| Enkephalin L \| 555.2693 \| HMDB0001045 \| 554.28 \| 2.243788 \| 1.21948 \| 1.8067 \| 0.045879 \| \| 2 \| 2,2,6,10,14-Pentamethylpentadecanoic acid \| 312.3028 \| HMDB0031318 \| 311.28 \| 7.361854 \| 1.401257 \| 2.3379 \| 0.029195 \| \| 3 \| Arachidyl alcohol \| 298.3236 \| HMDB0011619 \| 297.36 \| 18.66199 \| 1.471435 \| 1.9433 \| 0.00969 \| \| 4 \| 11,14,15-THETA \| 354.2406 \| HMDB0004694 \| 353.28 \| 7.53087 \| 1.748653 \| 2.8645 \| 0.006149 \| \| 5 \| (3beta,22E,24R)-5,8-Epidioxy-23-methylergosta-6,22-dien-3-ol \| 442.3447 \| HMDB0032668 \| 441.36 \| 13.5187 \| 1.491641 \| 3.8112 \| 0.005088 \| \| 6 \| alpha-Tocopherol \| 430.3811 \| HMDB0001893 \| 429.36 \| 22.48098 \| 1.146047 \| 1.6433 \| 0.028131 \| \| 7 \| Diisodityrosine \| 718.2486 \| HMDB0029798 \| 717.24 \| 20.31915 \| 1.236976 \| 2.994 \| 0.018822 \| \| 8 \| Vinaginsenoside R12 \| 672.4449 \| HMDB0040782 \| 671.4 \| 10.07921 \| 1.539396 \| 9.8315 \| 0.001916 \| \| 9 \| TG(20:5(5Z,8Z,11Z,14Z,17Z)/14:1(9Z)/o-18:0) \| 836.7258 \| HMDB0055716 \| 835.68 \| 18.77169 \| 1.076465 \| 1.7515 \| 0.038598 \| \| 10 \| (5alpha,6beta,14alpha,20R,22R)-5,6,14,20,27-Pentahydroxy-1-oxowith-24-enolide \| 506.288 \| HMDB0033198 \| 505.32 \| 18.4805 \| 1.480899 \| 0.69648 \| 0.025972 \| \| 11 \| Panaxynol linoleate \| 506.4124 \| HMDB0041177 \| 505.44 \| 18.46973 \| 1.279322 \| 0.74122 \| 0.048835 \| \| 12 \| Cerebronic acid \| 384.3603 \| HMDB0039540 \| 383.4 \| 2.754884 \| 1.094574 \| 1.5204 \| 0.042701 \| \| 13 \| Pyridoxal 5'-phosphate \| 247.0246 \| HMDB0001491 \| 246 \| 23.94002 \| 1.225184 \| 1.7472 \| 0.009053 \| |
| --- | --- | --- | --- | --- | --- | --- | --- | --- | --- | --- | --- | --- | --- | --- | --- | --- | --- | --- | --- | --- | --- | --- | --- | --- | --- | --- | --- | --- | --- | --- | --- | --- | --- | --- | --- | --- | --- | --- | --- | --- | --- | --- | --- | --- | --- | --- | --- | --- | --- | --- | --- | --- | --- | --- | --- | --- | --- | --- | --- | --- | --- | --- | --- | --- | --- | --- | --- | --- | --- | --- | --- | --- | --- | --- | --- | --- | --- | --- | --- | --- | --- | --- | --- | --- | --- | --- | --- | --- | --- | --- | --- | --- | --- | --- | --- | --- | --- | --- | --- | --- | --- | --- | --- | --- | --- | --- | --- | --- | --- | --- | --- | --- | --- | --- | --- | --- | --- | --- | --- | --- | --- | --- | --- | --- | --- | --- |

**Table 4: List of differential metabolites in serum metabolome profiling of HPAI H5N1 infection in chicken in positive ionisation mode**

| \| **No** \| **Name** \| **row m/z** \| **row retention time** \| **MonoMass** \| **HMDB** \| **VIP** \| **Fold change** \| **p-value** \| \| --- \| --- \| --- \| --- \| --- \| --- \| --- \| --- \| --- \| \| 1 \| 2,3-Diphosphoglyceric acid \| 267 \| 2.564203333 \| 265.9592695 \| HMDB0001294 \| 1.932081571 \| 1.6944 \| 0.0093659 \| \| 2 \| 2-Ethyl-4-methyl-1-pentanol \| 131.1600037 \| 23.92739417 \| 130.1357652 \| HMDB0013818 \| 1.270223183 \| 0.567 \| 0.041826 \| \| 3 \| Tetrahydrocortisone \| 365.2799988 \| 17.964655 \| 364.2249741 \| HMDB0000903 \| 1.611864423 \| 1.3973 \| 0.049672 \| \| 4 \| 2,3,6,7-Tetrahydrocyclopent[b]azepin-8(1H)-one \| 150.1199951 \| 1.969410833 \| 149.084064 \| HMDB0039662 \| 1.171597826 \| 0.70735 \| 0.045896 \| \| 5 \| 2,3-Dinor-TXB2 \| 343.2000122 \| 11.44549583 \| 342.2042387 \| HMDB0002904 \| 1.835692311 \| 0.46647 \| 0.0023993 \| \| 6 \| 3-(10-Heptadecenyl)phenol \| 331.3200073 \| 17.311825 \| 330.2922658 \| HMDB0038525 \| 1.430441813 \| 0.64209 \| 0.042397 \| \| 7 \| 3-(3,4-Dimethoxyphenyl)-2-propenoic acid \| 209.0399933 \| 15.66677583 \| 208.0735589 \| HMDB0034315 \| 1.992988837 \| 2.8885 \| 0.0035431 \| \| 8 \| 3-Methylpentanoic acid \| 117.1200027 \| 4.04804 \| 116.0837296 \| HMDB0033774 \| 1.160099524 \| 0.65662 \| 0.032923 \| \| 9 \| Deoxycytidine \| 228.1199951 \| 2.0361875 \| 227.0906059 \| HMDB0000014 \| 1.246793109 \| 0.74942 \| 0.020983 \| \| 10 \| 4,4-Dimethyl-5a-cholesta-8,24-dien-3-b-ol \| 413.3999939 \| 18.96603833 \| 412.3705162 \| HMDB0001286 \| 1.478186417 \| 3.2641 \| 0.047767 \| \| 11 \| 2,5-Dichloro-carboxymethylenebut-2-en-4-olide \| 208.9199982 \| 0.804975833 \| 207.933014 \| HMDB0060364 \| 1.813190307 \| 2.0717 \| 0.010758 \| \| 12 \| Quinolinic acid \| 168 \| 23.52214083 \| 167.0218577 \| HMDB0000232 \| 1.85507136 \| 2.0677 \| 0.015223 \| \| 13 \| N-Palmitoyl phenylalanine \| 404.2799988 \| 2.436285 \| 403.3086442 \| HMDB0062339 \| 2.115699573 \| 0.43367 \| 0.00034187 \| \| 14 \| L-Serine \| 106.0800018 \| 23.07412333 \| 105.0425931 \| HMDB0000187 \| 1.486334856 \| 0.52758 \| 0.026381 \| \| 15 \| N-Succinyl-2-amino-6-ketopimelate \| 290.0400085 \| 2.19007 \| 289.0797665 \| HMDB0012266 \| 2.339821147 \| 2.6929 \| 0.0014338 \| \| 16 \| gamma-Taraxastanonol \| 443.3999939 \| 15.46549417 \| 442.3810808 \| HMDB0033727 \| 1.2168961 \| 0.70262 \| 0.043053 \| \| 17 \| Lansic acid \| 471.3599854 \| 6.396463333 \| 470.33961 \| HMDB0036786 \| 1.499693422 \| 0.4184 \| 0.030472 \| \| 18 \| PE(22:0/22:6(4Z,7Z,10Z,13Z,16Z,19Z)) \| 848.6400146 \| 16.22459583 \| 847.6091052 \| HMDB0009507 \| 1.570465238 \| 0.56653 \| 0.0078317 \| \| 19 \| PE(22:6(4Z,7Z,10Z,13Z,16Z,19Z)/22:0) \| 848.6400146 \| 16.22459583 \| 847.6091052 \| HMDB0009699 \| 1.570465238 \| 0.56653 \| 0.0078317 \| \| 20 \| O-6-deoxy-a-L-galactopyranosyl-(1-&gt;2)-O-b-D-galactopyranosyl-(1-&gt;3)-2-(acetylamino)-2-deoxy-D-Galactose \| 530.1599731 \| 4.032840833 \| 529.2006695 \| HMDB0002060 \| 1.329806363 \| 0.64619 \| 0.029013 \| \| 21 \| Yucalexin B7 \| 301.2000122 \| 19.8840125 \| 300.2089301 \| HMDB0036711 \| 1.043886529 \| 0.80346 \| 0.0472 \| \| 22 \| L-Proline \| 116.0400009 \| 3.880399167 \| 115.0633285 \| HMDB0000162 \| 1.647722085 \| 1.8318 \| 0.045783 \| |
| --- | --- | --- | --- | --- | --- | --- | --- | --- | --- | --- | --- | --- | --- | --- | --- | --- | --- | --- | --- | --- | --- | --- | --- | --- | --- | --- | --- | --- | --- | --- | --- | --- | --- | --- | --- | --- | --- | --- | --- | --- | --- | --- | --- | --- | --- | --- | --- | --- | --- | --- | --- | --- | --- | --- | --- | --- | --- | --- | --- | --- | --- | --- | --- | --- | --- | --- | --- | --- | --- | --- | --- | --- | --- | --- | --- | --- | --- | --- | --- | --- | --- | --- | --- | --- | --- | --- | --- | --- | --- | --- | --- | --- | --- | --- | --- | --- | --- | --- | --- | --- | --- | --- | --- | --- | --- | --- | --- | --- | --- | --- | --- | --- | --- | --- | --- | --- | --- | --- | --- | --- | --- | --- | --- | --- | --- | --- | --- | --- | --- | --- | --- | --- | --- | --- | --- | --- | --- | --- | --- | --- | --- | --- | --- | --- | --- | --- | --- | --- | --- | --- | --- | --- | --- | --- | --- | --- | --- | --- | --- | --- | --- | --- | --- | --- | --- | --- | --- | --- | --- | --- | --- | --- | --- | --- | --- | --- | --- | --- | --- | --- | --- | --- | --- | --- | --- | --- | --- | --- | --- | --- | --- | --- | --- | --- | --- | --- | --- | --- | --- | --- | --- | --- | --- | --- | --- | --- | --- |

**Table 5: List of differential metabolites in serum metabolome profiling of HPAI H5N1 infection in chicken in negative ionisation mode**

| \| No \| **Name** \| **row m/z** \| **row retention time** \| **MonoMass** \| **HMDB** \| **VIP** \| **Fold Change** \| **p-value** \| \| --- \| --- \| --- \| --- \| --- \| --- \| --- \| --- \| --- \| \| 1 \| N-Succinyl-2-amino-6-ketopimelate \| 288.119995 \| 2.56415083 \| 289.079767 \| HMDB0012266 \| 1.29775319 \| 1.7653 \| 0.030998 \| \| 2 \| 3-(3,5-dihydroxyphenyl)propanoic acid \| 181.080002 \| 3.6472425 \| 182.057909 \| HMDB0125533 \| 1.54080569 \| 0.60581 \| 0.044656 \| \| 3 \| PE(18:3(6Z,9Z,12Z)/P-16:0) \| 696.479981 \| 14.1964408 \| 697.50464 \| HMDB0009147 \| 1.35288087 \| 1.8656 \| 0.025999 \| \| 4 \| PE(18:3(9Z,12Z,15Z)/P-16:0) \| 696.479981 \| 14.1964408 \| 697.50464 \| HMDB0009180 \| 1.35288087 \| 1.8656 \| 0.025999 \| \| 5 \| Bornyl formate \| 181.080002 \| 3.31222917 \| 182.13068 \| HMDB0038245 \| 1.2346913 \| 1.5525 \| 0.045829 \| \| 6 \| Perillic acid \| 165.119995 \| 9.41229917 \| 166.09938 \| HMDB0004586 \| 1.46071339 \| 0.46491 \| 0.04355 \| \| 7 \| alpha-Methylstyrene \| 117.120003 \| 6.34231083 \| 118.07825 \| HMDB0059899 \| 1.73244614 \| 0.53117 \| 0.04208 \| \| 8 \| Momordicilin \| 539.400024 \| 1.44271917 \| 540.454246 \| HMDB0030896 \| 1.35939211 \| 2.927 \| 0.044871 \| \| 9 \| Methylthiomethyl butyrate \| 147 \| 4.27925833 \| 148.0558 \| HMDB0038299 \| 1.83944115 \| 2.4092 \| 0.028357 \| \| 10 \| Estrone glucuronide \| 445.200012 \| 8.43304833 \| 446.194068 \| HMDB0004483 \| 1.73209339 \| 1.708 \| 0.015334 \| \| 11 \| Cycloviolaxanthin \| 599.400024 \| 12.7481758 \| 600.41786 \| HMDB0038106 \| 1.78606094 \| 1.9201 \| 0.012226 \| \| 12 \| Guanidoacetic acid \| 116.040001 \| 21.1237717 \| 117.053827 \| HMDB0000128 \| 1.9775463 \| 0.29744 \| 0.0057791 \| \| 13 \| Taurohyocholate \| 514.320007 \| 12.7322283 \| 515.291674 \| HMDB0011637 \| 1.60727278 \| 1.8419 \| 0.035303 \| \| 14 \| Kaurenoic acid methyl ester \| 315.23999 \| 4.2757775 \| 316.24023 \| HMDB0036729 \| 1.93763184 \| 1.7172 \| 0.010647 \| \| 15 \| NADP \| 743.039978 \| 20.1167258 \| 744.083277 \| HMDB0000217 \| 1.77326167 \| 0.4948 \| 0.02293 \| |
| --- | --- | --- | --- | --- | --- | --- | --- | --- | --- | --- | --- | --- | --- | --- | --- | --- | --- | --- | --- | --- | --- | --- | --- | --- | --- | --- | --- | --- | --- | --- | --- | --- | --- | --- | --- | --- | --- | --- | --- | --- | --- | --- | --- | --- | --- | --- | --- | --- | --- | --- | --- | --- | --- | --- | --- | --- | --- | --- | --- | --- | --- | --- | --- | --- | --- | --- | --- | --- | --- | --- | --- | --- | --- | --- | --- | --- | --- | --- | --- | --- | --- | --- | --- | --- | --- | --- | --- | --- | --- | --- | --- | --- | --- | --- | --- | --- | --- | --- | --- | --- | --- | --- | --- | --- | --- | --- | --- | --- | --- | --- | --- | --- | --- | --- | --- | --- | --- | --- | --- | --- | --- | --- | --- | --- | --- | --- | --- | --- | --- | --- | --- | --- | --- | --- | --- | --- | --- | --- | --- | --- | --- | --- | --- | --- |

**Table 6: Pathway enrichment analysis of lung tissue sample**

| **No** | **Metabolite pathway** | **Total** | **Hits** | **p-value** |
| --- | --- | --- | --- | --- |
| 1 | Sphingolipid Metabolism | 40 | 2 | 0.0211 |
| 2 | Tryptophan Metabolism | 59 | 2 | 0.0439 |
| 3 | Homocysteine Degradation | 9 | 1 | 0.0528 |
| 4 | Malate-Aspartate Shuttle | 10 | 1 | 0.0585 |
| 5 | Taurine and Hypotaurine Metabolism | 12 | 1 | 0.0699 |
| 6 | Glucose-Alanine Cycle | 13 | 1 | 0.0755 |
| 7 | Alanine Metabolism | 17 | 1 | 0.0978 |
| 8 | Spermidine and Spermine Biosynthesis | 18 | 1 | 0.103 |
| 9 | Vitamin B6 Metabolism | 19 | 1 | 0.109 |
| 10 | Catecholamine Biosynthesis | 20 | 1 | 0.114 |
| 11 | Threonine and 2-Oxobutanoate Degradation | 20 | 1 | 0.114 |
| 12 | Carnitine Synthesis | 22 | 1 | 0.125 |
| 13 | Cysteine Metabolism | 26 | 1 | 0.146 |
| 14 | Phenylalanine and Tyrosine Metabolism | 27 | 1 | 0.152 |
| 15 | Selenoamino Acid Metabolism | 27 | 1 | 0.152 |
| 16 | Urea Cycle | 28 | 1 | 0.157 |
| 17 | Folate Metabolism | 29 | 1 | 0.162 |
| 18 | Lysine Degradation | 30 | 1 | 0.167 |
| 19 | Ammonia Recycling | 31 | 1 | 0.172 |
| 20 | Starch and Sucrose Metabolism | 31 | 1 | 0.172 |
| 21 | Beta-Alanine Metabolism | 34 | 1 | 0.188 |
| 22 | Aspartate Metabolism | 35 | 1 | 0.193 |
| 23 | Porphyrin Metabolism | 40 | 1 | 0.217 |
| 24 | Propanoate Metabolism | 42 | 1 | 0.227 |
| 25 | Methionine Metabolism | 42 | 1 | 0.227 |
| 26 | Histidine Metabolism | 42 | 1 | 0.227 |
| 27 | Glutamate Metabolism | 48 | 1 | 0.256 |
| 28 | Arginine and Proline Metabolism | 52 | 1 | 0.274 |
| 29 | Pyrimidine Metabolism | 57 | 1 | 0.297 |
| 30 | Glycine and Serine Metabolism | 59 | 1 | 0.306 |
| 31 | Valine, Leucine and Isoleucine Degradation | 59 | 1 | 0.306 |
| 32 | Arachidonic Acid Metabolism | 67 | 1 | 0.341 |
| 33 | Tyrosine Metabolism | 70 | 1 | 0.353 |
| 34 | Purine Metabolism | 73 | 1 | 0.366 |

**Table 7: Pathway enrichment analysis overview of serum**

| **No** | **Metabolic Pathway** | **Total** | **Hits** | **p-value** |
| --- | --- | --- | --- | --- |
| 1 | Arginine and Proline Metabolism | 52 | 3 | 0.0122 |
| 2 | Estrone Metabolism | 24 | 2 | 0.022 |
| 3 | Nicotinate and Nicotinamide Metabolism | 35 | 2 | 0.0447 |
| 4 | Sphingolipid Metabolism | 40 | 2 | 0.0571 |
| 5 | Steroidogenesis | 43 | 2 | 0.0651 |
| 6 | Steroid Biosynthesis | 48 | 2 | 0.0791 |
| 7 | Homocysteine Degradation | 9 | 1 | 0.0866 |
| 8 | Degradation of Superoxides | 11 | 1 | 0.105 |
| 9 | Pyrimidine Metabolism | 57 | 2 | 0.107 |
| 10 | Glycine and Serine Metabolism | 59 | 2 | 0.113 |
| 11 | Tryptophan Metabolism | 59 | 2 | 0.113 |
| 12 | Phosphatidylethanolamine Biosynthesis | 12 | 1 | 0.114 |
| 13 | Glucose-Alanine Cycle | 13 | 1 | 0.123 |
| 14 | Vitamin K Metabolism | 13 | 1 | 0.123 |
| 15 | Ethanol Degradation | 19 | 1 | 0.175 |
| 16 | Glutathione Metabolism | 20 | 1 | 0.183 |
| 17 | Ubiquinone Biosynthesis | 20 | 1 | 0.183 |
| 18 | Transfer of Acetyl Groups into Mitochondria | 22 | 1 | 0.2 |
| 19 | Glycolysis | 23 | 1 | 0.208 |
| 20 | Androstenedione Metabolism | 24 | 1 | 0.216 |
| 21 | Glycerolipid Metabolism | 25 | 1 | 0.224 |
| 22 | Phytanic Acid Peroxisomal Oxidation | 26 | 1 | 0.232 |
| 23 | Plasmalogen Synthesis | 26 | 1 | 0.232 |
| 24 | Selenoamino Acid Metabolism | 27 | 1 | 0.24 |
| 25 | Pterine Biosynthesis | 28 | 1 | 0.248 |

**Table 8: ROC (Receiver Operating Characteristic) curve analysis of lung metabolites**

AUC – Area Under Curve

| **No** | **Metabolite** | **AUC** |
| --- | --- | --- |
| 1 | DG(20:3(8Z,11Z,14Z)/24:0/0:0) | 0.92593 |
| 2 | DG(22:1(13Z)/22:2(13Z,16Z)/0:0) | 0.92593 |
| 3 | DG(24:0/20:3(5Z,8Z,11Z)/0:0) | 0.92593 |
| 4 | DG(24:1(15Z)/20:2(11Z,14Z)/0:0) | 0.92593 |
| 5 | PS(16:1(9Z)/16:1(9Z)) | 0.9008 |
| 6 | (5alpha,6beta,14alpha,20R,22R)-5,6,14,20,27-Pentahydroxy-1-oxowith-24-enolide | 0.88889 |
| 7 | Panaxynol linoleate | 0.88889 |
| 8 | Vinaginsenoside R12 | 0.87654 |
| 9 | Sphingosine | 0.83916 |
| 10 | 11,14,15-THETA | 0.76543 |
| 11 | 1alpha,3beta,22R-Trihydroxyergosta-5,24E-dien-26-oic acid 3-O-b-D-glucoside 26-O-b-D-glucosyl ester | 0.76543 |
| 12 | 3,4-Methylenesebacic acid | 0.76543 |
| 13 | N-(2-Hydroxyethyl)-morpholine | 0.74074 |
| 14 | 11'-Carboxy-gamma-tocotrienol | 0.7284 |
| 15 | Serylarginine | 0.7284 |
| 16 | (3beta,22E,24R)-5,8-Epidioxy-23-methylergosta-6,22-dien-3-ol | 0.71605 |
| 17 | gamma-L-Glutamyl-L-methionine sulfoxide | 0.7037 |
| 18 | Rubrofusarin 6-[glucosyl-(1->3)-glucosyl-(1->6)-glucoside] | 0.7037 |
| 19 | Furanodienone | 0.7037 |
| 20 | Indoleacetaldehyde | 0.7037 |
| 21 | Arachidyl alcohol | 0.7037 |
| 22 | TG(20:5(5Z,8Z,11Z,14Z,17Z)/14:1(9Z)/o-18:0) | 0.7037 |
| 23 | 2-Pyrrolidineacetic acid | 0.69136 |
| 24 | Pyridoxal 5'-phosphate | 0.69136 |
| 25 | Cytidine | 0.67901 |
| 26 | 2,2,6,10,14-Pentamethylpentadecanoic acid | 0.66667 |
| 27 | 2-Hydroxyestradiol | 0.65432 |
| 28 | 2-(1,2,3,4-Tetrahydroxybutyl)-6-(2,3,4-trihydroxybutyl)pyrazine | 0.64198 |
| 29 | Guanine | 0.62963 |
| 30 | Enkephalin L | 0.62963 |
| 31 | alpha-Tocopherol | 0.62963 |
| 32 | Cyanidin 3-(6''-dioxalylglucoside) | 0.60494 |
| 33 | 1,3-Diphenyl-1-propanone | 0.60494 |
| 34 | N-Arachidonoyl glycine | 0.60494 |
| 35 | N-arachidonoylglycinate | 0.60494 |
| 36 | Diisodityrosine | 0.60494 |
| 37 | Heptyl heptanoate | 0.59259 |
| 38 | TG(14:0/20:0/o-18:0) | 0.58025 |
| 39 | Urothion | 0.58025 |
| 40 | Cerebronic acid | 0.5679 |
| 41 | Psychosine sulfate | 0.55556 |
| 42 | TG(16:0/22:1(13Z)/o-18:0) | 0.54321 |
| 43 | N-a-Acetyl-L-arginine | 0.53086 |
| 44 | Tetradecanol | 0.51852 |

**Table 9: ROC (Receiver Operating Characteristic) curve analysis of serum metabolites**

| **No** | **Metabolite** | **AUC** |
| --- | --- | --- |
| 1 | 3-(3,4-Dimethoxyphenyl)-2-propenoic acid | 0.938 |
| 2 | Cycloviolaxanthin | 0.92593 |
| 3 | 2,3-Diphosphoglyceric acid | 0.9142 |
| 4 | Kaurenoic acid methyl ester | 0.90123 |
| 5 | 4,4-Dimethyl-5a-cholesta-8,24-dien-3-b-ol | 0.90123 |
| 6 | Quinolinic acid | 0.88889 |
| 7 | N-Palmitoyl phenylalanine | 0.88889 |
| 8 | Tetrahydrocortisone | 0.87654 |
| 9 | 2,3-Dinor-TXB2 | 0.8642 |
| 10 | N-Succinyl-2-amino-6-ketopimelate | 0.8642 |
| 11 | Guanidoacetic acid | 0.8642 |
| 12 | 2,5-Dichloro-carboxymethylenebut-2-en-4-olide | 0.85185 |
| 13 | PE(18:3(6Z,9Z,12Z)/P-16:0) | 0.83951 |
| 14 | PE(18:3(9Z,12Z,15Z)/P-16:0) | 0.83951 |
| 15 | Methylthiomethyl butyrate | 0.83951 |
| 16 | Estrone glucuronide | 0.82716 |
| 17 | L-Serine | 0.81481 |
| 18 | Bornyl formate | 0.80247 |
| 19 | Momordicilin | 0.80247 |
| 20 | L-Proline | 0.80247 |
| 21 | 3-Methylpentanoic acid | 0.79012 |
| 22 | Taurohyocholate | 0.77778 |
| 23 | PE(22:0/22:6(4Z,7Z,10Z,13Z,16Z,19Z)) | 0.76543 |
| 24 | PE(22:6(4Z,7Z,10Z,13Z,16Z,19Z)/22:0) | 0.75309 |
| 25 | 3-(3,5-dihydroxyphenyl)propanoic acid | 0.75309 |
| 26 | NADP | 0.7284 |
| 27 | 2-Ethyl-4-methyl-1-pentanol | 0.7284 |
| 28 | Perillic acid | 0.71605 |
| 29 | alpha-Methylstyrene | 0.71605 |
| 30 | Lansic acid | 0.71605 |
| 31 | O-6-deoxy-a-L-galactopyranosyl-(1->2)-O-b-D-galactopyranosyl-(1->3)-2-(acetylamino)-2-deoxy-D-Galactose | 0.7037 |
| 32 | gamma-Taraxastanonol | 0.7037 |
| 33 | 3-(10-Heptadecenyl)phenol | 0.69136 |
| 34 | Deoxycytidine | 0.67901 |
| 35 | Yucalexin B7 | 0.66667 |
| 36 | 2,3,6,7-Tetrahydrocyclopent[b]azepin-8(1H)-one | 0.60494 |
